# Postbiotics from *Saccharomyces cerevisiae* fermentation stabilize rumen solids microbiota and promote microbial network interactions and diversity of hub taxa during grain-based subacute ruminal acidosis (SARA) challenges in lactating dairy cows

**DOI:** 10.3389/fmicb.2024.1409659

**Published:** 2024-08-16

**Authors:** Junfei Guo, Zhengxiao Zhang, Le Luo Guan, Mi Zhou, Ilkyu Yoon, Ehsan Khafipour, Jan C. Plaizier

**Affiliations:** ^1^Department of Animal Science, University of Manitoba, Winnipeg, MB, Canada; ^2^Department of Agriculture, Food and Nutrition, University of Alberta, Edmonton, AB, Canada; ^3^Diamond V, Cedar Rapids, IA, United States

**Keywords:** dairy cows, postbiotics, *Saccharomyces cerevisiae* fermentation product, SARA, rumen solids microbiota

## Abstract

**Background:**

High-yielding dairy cows are commonly fed high-grain rations. However, this can cause subacute ruminal acidosis (SARA), a metabolic disorder in dairy cows that is usually accompanied by dysbiosis of the rumen microbiome. Postbiotics that contain functional metabolites provide a competitive niche for influential members of the rumen microbiome, may stabilize and promote their populations, and, therefore, may attenuate the adverse effects of SARA.

**Methods:**

This study used a total of 32 rumen-cannulated lactating dairy cows, which were randomly assigned into four treatments: no SCFP (control), 14 g/d Original XPC (SCFPa), 19 g/d NutriTek (SCFPb-1X), and 38 g/d NutriTek (SCFPb-2X) (Diamond V, Cedar Rapids, IA) from 4 weeks before until 12 weeks after parturition. Grain-based SARA challenges were conducted during week 5 (SARA1) and week 8 (SARA2) after parturition by replacing 20% dry matter of the base total mixed ration (TMR) with pellets containing 50% ground barley and 50% ground wheat. The DNA of rumen solids digesta was extracted and subjected to V3-V4 16S rRNA gene sequencing. The characteristics of rumen solids microbiota were compared between non-SARA (Pre-SARA1, week 4; Post-SARA1, week 7; and Post-SARA2, weeks 10 and 12) and SARA stages (SARA1/1, SARA1/2, SARA2/1, SARA2/2), as well as among treatments.

**Results:**

Both SARA challenges reduced the richness and diversity of the microbiota and the relative abundances of the phylum Fibrobacteres. Supplementation with SCFP promoted the growth of several fibrolytic bacteria, including Lachnospiraceae UCG-009, *Treponema*, unclassified Lachnospiraceae, and unclassified Ruminococcaceae during the SARA challenges. These challenges also reduced the positive interactions and the numbers of hub taxa in the microbiota. The SCFPb treatment increased positive interactions among microbial members of the solids digesta and the number of hub taxa during the SARA and non-SARA stages. The SCFPb-2X treatment prevented changes in the network characteristics, including the number of components, clustering coefficient, modularity, positive edge percentage, and edge density of the microbiota during SARA challenges. These challenges reduced predicted carbohydrate and nitrogen metabolism in microbiota, whereas SCFP supplementation attenuated those reductions.

**Conclusions:**

Supplementation with SCFP, especially the SCFPb-2X attenuated the adverse effects of grain-based SARA on the diversity and predicted functionality of rumen solids microbiota.

## 1 Introduction

High-grain diets are widely fed to high milk-producing dairy cows to meet their high energy requirements. These diets can result in the accumulation of volatile fatty acids (VFAs) such as acetate, propionate, and butyrate from rumen fermentation and reduced rumen buffering, resulting in rumen pH depressions (Plaizier et al., 2012). When the rumen pH remains for extended periods, e.g., more than 180 min/d, below 5.6, it can result in subacute ruminal acidosis (SARA) (Cooper et al., 1997; Gozho et al., 2005; Plaizier et al., 2008). Further decline of rumen pH below 5.0 results in acute ruminal acidosis, which involves the accumulation of lactate acid in the rumen (Zebeli and Metzler-Zebeli, 2012; Plaizier et al., 2018, 2022). SARA is especially prevalent during early and mid-lactation due to switches from high-forage to high-grain diets (Kleen et al., 2003; Plaizier et al., 2008; Valente et al., 2017). A depressed rumen pH can interrupt the barrier function of the rumen epithelium. This disruption causes the release of immunogenic compounds, such as lipopolysaccharides (LPS), from gram-negative bacteria and their translocation from the rumen into the systemic circulation (Khafipour et al., 2009a; Plaizier et al., 2022). LPS binds to toll-like receptor 4 on cytomembranes and triggers the release of the pro-inflammatory cytokines and the production of acute phase proteins in the liver, causing a systemic immune response in dairy cows (Eckel and Ametaj, 2016; Li et al., 2016).

Rumen bacteria produce a wide array of enzymes to break down structural and non-structural carbohydrates, protein and peptides, and triglycerides into absorbable substrates that can then be utilized by dairy cows (Russell and Rychlik, 2001; Firkins and Yu, 2015; Plaizier et al., 2018; Gruninger et al., 2019). The composition and functionality of the rumen microbiota can be changed because of changes in the diet and/or the availability of substrates for microbiota since some microbes can take advantage of the newly available substrates while others cannot (Khafipour et al., 2009b; Mao et al., 2013; Agler et al., 2016). The colonization of microbes in the rumen relies on other members of the community, such that their competitive advantage can increase when others provide them with metabolites. Consequently, direct interactions among microbes play an important role in determining the rumen microbiome structure (Faust and Raes, 2012, 2016; Agler et al., 2016; Manirajan et al., 2018).

To predict the microbe-microbe interactions from repeated measurements of their presence or abundance, co-occurrence analysis is commonly used as microbial network inference (Faust and Raes, 2016). From microbial interactions, “hub taxa,” which are observed as the most interactive OTUs/ASVs in a microbial network (e.g., having the most positive or negative connections with other members), are key in shaping the microbiome structures. These hub taxa control the abundance of many other microorganisms as they can promote or suppress the growth and diversity of those microbes (Faust and Raes, 2012; Agler et al., 2016; Manirajan et al., 2018; Derakhshani et al., 2020). Therefore, assessing the shifts in microbial connections and hub taxa aids in better understanding the effect of the diet or host phenotype on the entire microbial community. The abundance of several hub taxa, such as fibrolytic bacteria that are pH sensitive, decreases when the rumen pH declines (Nagaraja and Titgemeyer, 2007; Zhou et al., 2015). Accordingly, SARA affects the composition and functionality of the rumen microbiota by reducing its richness, evenness, and the relative abundances of several hub taxa, including those of fibrolytic bacteria (Mao et al., 2013; Brede et al., 2020). Although SARA negatively affects milk production, animal health, and welfare, its clinical symptoms can be easily unnoticed. Hence, it is critical to prevent SARA and attenuate its adverse effects.

Several strategies have been developed to attenuate the negative effect of SARA, such as supplementing diets with buffers, ionophores, prebiotics, probiotics, and postbiotics. Among these, postbiotics have received more attention in recent years. Postbiotics are “a preparation of inanimate microorganisms and/or their components that confers a health benefit on the host” (Salminen et al., 2021). As such, a postbiotic formulation may contain microbial cell components, microbial metabolites produced through the anabolic activity of microbes, and intermediate or end-products of microbial fermentation produced through the catabolic activity of microbes (Duysburgh et al., 2024). Some of these metabolites and compounds affect the cross-feeding patterns among microbes, while others act as signaling molecules or neurotransmitters affecting the microbe-microbe or microbe-host interactions (Salminen et al., 2021). As a result, postbiotics potentially have a wider variety of modes of action compared to other biotic strategies (Salminen et al., 2021). Several studies have shown that supplementation with postbiotics reduces inflammation, promotes milk production, improves the efficiency of feed utilization in lactating dairy cows and calves, and stabilizes rumen pH and rumen fermentation during high-grain feeding (Acharya et al., 2017; Mahmoud et al., 2020; Vailati-Riboni et al., 2021; Guo et al., 2022).

Rumen bacteria comprises a large part (>95%) of the rumen microbiota, which can be divided into different subpopulations, such as rumen liquid microbiota and rumen solids microbiota, based on their adhering and colonization sites (Zhou et al., 2015). Previous studies have mostly assessed the microbiota changes only in rumen liquid fraction due to easier sampling methods, and few have reported shifts in the rumen solids microbial community, especially when cows were exposed to metabolic stressors such as SARA (Martin et al., 2001; Michalet-Doreau et al., 2001; Petri et al., 2013; McCann et al., 2016; Ji et al., 2017; Brede et al., 2020). Additionally, the dynamic changes of the rumen bacteria during early- and mid-lactation when supplemented with postbiotics were rarely examined in previous studies.

In this study, we tested two postbiotics from *Saccharomyces cerevisiae* fermentation (SCFP) (Original XPC and NutriTek, Diamond V, Cedar Rapids, IA), where NutriTek contained higher concentrations of antioxidants and polyphenol compounds among other metabolites compared to XPC. In our companion papers, we reported that NutriTek was more efficient in stabilizing rumen pH (Khalouei et al., 2020) and rumen liquid microbiota (Guo et al., 2024) and further reduced inflammation in dairy cows during SARA compared to XPC (Guo et al., 2022). Therefore, this study aimed to investigate: (1) the effect of grain-based SARA challenges on microbial diversity and predicted functionalities of the rumen solids microbiota, and (2) the effects of two SCFPs on composition, functionality, and interaction among rumen solids microbes in lactating dairy cows subjected to repeated grain-based SARA challenges.

## 2 Materials and methods

The protocol used in this study was approved by the University of Manitoba Animal Care Committee (Protocol # F14-038) and followed the guidelines of the Canadian Council for Animal Care (CCAC, 1993).

### 2.1 Animals, diet, and experimental design

Complete details of the animal management and experimental design are described by Guo et al. (2022). Briefly, a total of 32 rumen-cannulated lactating dairy cows were used in a randomized complete block design with 8 blocks. Cows were placed in blocks based on their parity, previous milk yield, and expected calving date. Cows were fitted with cannulas approximately 12 weeks before calving and had fully recovered before this experiment started, as confirmed by veterinarians. Within each block, cows were randomly assigned to one of four treatments: a basal diet without supplementation (control) or the basal diet supplemented with 14 g/d Diamond V Original XPC (SCFPa, Diamond V), 19 g/d NutriTek (SCFPb-1X, Diamond V), or 38 g/d NutriTek (SCFPb-2X, Diamond V) mixed with 140, 126, 121, and 102 g/d of ground corn, respectively.

The SCFP was supplemented once daily as a top-dress after morning delivery of the diet. A detailed description of the feed sample collection and analyses has been reported by Khalouei et al. (2020). The chemical and nutrient composition of diets are shown in companion papers (Khalouei et al., 2020; Guo et al., 2022). Briefly, cows were fed individually with a prepartum diet containing 38.7% dry matter (DM) of neutral detergent fiber (NDF), 15.5% crude protein (CP), and 17.6% starch (DM basis) from 4 weeks before parturition. They were then switched to a lactation diet containing 34.9% DM NDF, 7.9% DM CP, and 18.6% DM starch until 12 weeks after parturition, except for SARA challenge weeks. On weeks 5 and 8, SARA challenges were conducted (SARA1, SARA2) by gradually replacing 20% DM of the base TMR with pellets containing 50% ground barley and 50% ground wheat over 3 days. The SARA induction diet contained 28.2% DM NDF, 17.2% DM CP, and 27.9% DM starch. The replacement was completed gradually over 3 days before SARA1 and SARA2 weeks. During the 2 weeks between the SARA challenges, cows returned to the base TMR and the treatment supplementations to wash out the first SARA effect. Cows were fed the TMR *ad libitum* once daily at 0900 h, with feed refusal allowances of 5%−10%. Cows were housed in individual stalls and had free access to fresh water during the whole experiment.

### 2.2 Sample collection and processing

#### 2.2.1 Sample collection

Rumen digesta samples were taken 6 h after feed delivery once weekly on weeks −4, −1, 1, 3, 4 (Pre-SARA1), 7 (Post-SARA1), 10 (Post-SARA2), and 12 (Post-SARA2) from five sites including cranial, caudal, dorsal, caudal ventral, and caudal dorsal of the rumen. On weeks 5 (SARA 1) and 8 (SARA2), rumen samples were taken on the second and fifth days of these weeks (timepoints were indicated as SARA1/1, SARA1/2, SARA2/1, SARA2/2). After that, rumen solids and liquid digesta were separated using a Bodum coffee filter plunger (Bodum Inc. Triengen, Switzerland) as described in our companion paper (Guo et al., 2022). An approximately 8 g of rumen solids sample was aliquoted into plastic bags, snapped frozen immediately in liquid nitrogen, and stored at −80°C for further microbial analysis.

#### 2.2.2 DNA extraction

Genomic DNA of rumen solids was extracted using *Quick*-DNA ZR Fecal/Soil DNA kits (D6010; Zymo Research Corp., Orange, CA) following the manufacturer's procedures as described previously (Guo et al., 2024). The procedure included a 2-min bead-beating step at 1750 strokes per minute using a Geno/Grinder equipment (2010, SPEX SamplePrep, Metuchen, NJ, USA). Genomic DNA was quantified using a NanoDrop 2000 spectrophotometer (Thermo Scientific, Waltham, MA, USA) and was normalized to 20 ng/μl followed by MiSeq Illumina sequencing. The DNA samples were quality checked by gel electrophoresis and PCR amplification of the 16S rRNA gene using universal primers 27F (5′-GAAGAGTTTGATCATGGCTCAG-3′) and 342R (5′-CTGCTGCCTCCCGTAG-3′) as described previously (Khafipour et al., 2009b). Amplicons were verified by agarose gel electrophoresis.

#### 2.2.3 PCR amplification and construction of sequencing libraries

These procedures were described by Guo et al. (2024) for rumen liquid digesta samples. The PCR was targeted to amplify the V3-V4 regions of the bacterial 16S rRNA genes using modified F338/R306 primers. The PCR reactions included an initial denaturing step at 94°C for 3 min, followed by 32 amplification cycles at 94°C for 30 s, 55°C for 20 s, and 72°C for 20 s, with a final extension step at 72°C for 5 min in an Eppendorf Mastercycler pro-S (Eppendorf, Hamburg, Germany). Subsequently, the sequencing library was generated and sequenced using a MiSeq Reagent Kit V3 (600-cycle; Illumina, San Diego, CA, USA) at the Gut Microbiome and Large Animal Biosecurity Laboratories, Department of Animal Science, University of Manitoba, Winnipeg, MB, Canada.

### 2.3 Bioinformatics and statistical analyses

#### 2.3.1 Bioinformatics analyses

Bioinformatics and statistical analysis were performed as described by Guo et al. (2024). Briefly, the DADA2 algorithm was used for quality control, and the feature table was constructed. Sequences were assigned into amplicon sequence variants (ASVs), and taxonomy was classified with QIIME 2 2023.2 (Bolyen et al., 2019). Only samples with sequencing depth >6,072 were kept. Community α-diversity (Shannon's diversity index, Observed Features index, Faith's Phylogenetic Diversity (PD) index, and Pielou's Evenness index) and β-diversity (Jaccard distance, Bray-Curtis distance, unweighted UniFrac distance and weighted UniFrac distance indices) metrics were computed using QIIME 2 default scripts, at an even depth per sample. Non-metric multidimensional scaling (nMDS) was applied on the resulting Bray-Curtis distance matrices to generate two-dimensional plots using default settings of the PRIMER-E software ver. 7.0.17 (Clarke and Gorley, 2015).

#### 2.3.2 Statistical analyses of rumen solids microbiota composition and diversity

Similar to that described by Guo et al. (2024), the univariate procedure of SAS (v 9.4, SAS Institute Inc., Cary, NC, USA) was used for testing the normality of residuals for α-diversity and the relative abundances of bacteria analyses. If the residuals were not normal, the data was log or Box-Cox transformed for normalization before being subjected to the mixed procedure of SAS as described in the results tables. The block was considered a random effect, the stage was repeated measure, and parity and treatment were fixed effects. The effect of parity was removed from the model when its *p* > 0.10. All pairwise comparisons between the groups were tested using the Tukey studentized range adjustment. Contrast comparisons were made between SARA 1 and SARA 2 stages, as well as control and SCFP, SCFPa and SCFPb, and SCFPb-1X and SCFPb-2X. Significant effects were considered at p < 0.05, and tendencies at 0.05 ≤ p < 0.1 were discussed. Data were presented as means from the original data in this study.

Permutational multivariate analysis of variance (PERMANOVA; implemented in PRIMER-E software v.7.0.17) (Clarke and Gorley, 2015) was used to detect significant differences between β-diversity metrics of bacterial communities as described by Guo et al. (2024). Non-metric multidimensional scaling (nMDS) was applied to the resulting distance matrices to generate two-dimensional plots using the default settings of the PRIMERE. Further to PERMANOVA, permutational multivariate analysis of dispersion (PERMDISP) was performed in PRIMER-E to detect the homogeneity of the dispersions among treatments and stages in rumen solids microbiota (Clarke and Gorley, 2015).

Compositional dynamics of solids digesta bacterial communities of the rumen were assessed using the Metagenomic Longitudinal Differential Abundance (MetaLonDA) method with edgeR package in R (Metwally et al., 2018), similarly as for rumen liquid microbiota (Guo et al., 2024). Taxonomic profiles were normalized with cumulative sum scaling (CSS), and pairwise comparisons were conducted between control vs. SCFPb-2X, control vs. SCFPb-1X, and control vs. SCFPa treatments. Within each comparison, the longitudinal profiles were fitted with a negative binomial smoothing spline. Significant time intervals were identified when *p* < 0.05 after multiple testing corrections by Benjamini and Hochberg (1995). Data were presented both at the phylum and genus levels.

#### 2.3.3 Functional prediction of rumen solids microbiota

Predicted functions of the rumen solids microbiome, including amino acid, lipid, and carbohydrate metabolic pathways, were assessed by the Phylogenetic Investigation of Communities by Reconstruction of Unobserved States (PICRUSt; CowPI) (Wilkinson et al., 2018). The differences between the control and SCFP supplementation treatment groups during SARA challenges were analyzed by STAMP v2.1.3 (Parks et al., 2014) using an ANOVA test. Significant differences were adjusted using the Tukey-Kramer test and identified when *p* < 0.05 after multiple testing corrections by Benjamini-Hochberg's false discovery rate (FDR) correction.

#### 2.3.4 Co-occurrence analysis

Correlation network analysis (CoNet) (Derakhshani et al., 2018; Zhang et al., 2018) was used to determine microbial co-occurrence/mutual-exclusion relationships among rumen solids microbial members at the genus level under SARA (SARA1 and SARA2) and non-SARA (pre-SARA1, post-SARA1, post-SARA2) conditions. To determine the influential capacity of bacterial taxa (Trosvik and de Muinck, 2015), their degree of connectedness was calculated by dividing the total number of edges (connections) observed for each phylum by its relative abundance in the community. Hub ASVs in each niche were identified when they had more than 15 edges with other members in the community. The correlation between hub ASVs and biodiversity metrics, rumen VFAs, and ammonia concentrations was conducted by Spearman's rank correlation coefficient (rho) and Corrplot package of R (Wei et al., 2017).

The NetCoMi (network construction and comparison for microbiome data) was used to determine if associations between paired microbes and the overall network structure differed among treatments (Peschel et al., 2021). The same data filtering and normalization were used as in CoNet. Subsequently, group comparisons were conducted by calculating network summary characteristics, including the number of components, clustering coefficients, modularity, positive edge percentage, edge density, and natural connectivity. The network comparison module assessed the overall network structure differences between the treatment groups and experimental periods, using permutation tests with 1,000 replicates, and the differential associations with an alpha of 0.05 were selected (Peschel et al., 2021).

## 3 Results

Three companion papers reported the effects of SCFPs and SARA on feed intake, milk production, rumen fermentation, and nutrient digestibilities (Khalouei et al., 2020), bacterial endotoxin concentrations and markers of inflammation (Guo et al., 2022), and composition and functionality of microbiota in rumen liquid digesta in lactating dairy cows (Guo et al., 2024). In this study, we detected the effects of SCFPs and SARA on the characteristics of rumen solids microbiota.

### 3.1 Alpha- and beta-diversity dynamics of rumen solids microbiota

**Figure 1:**
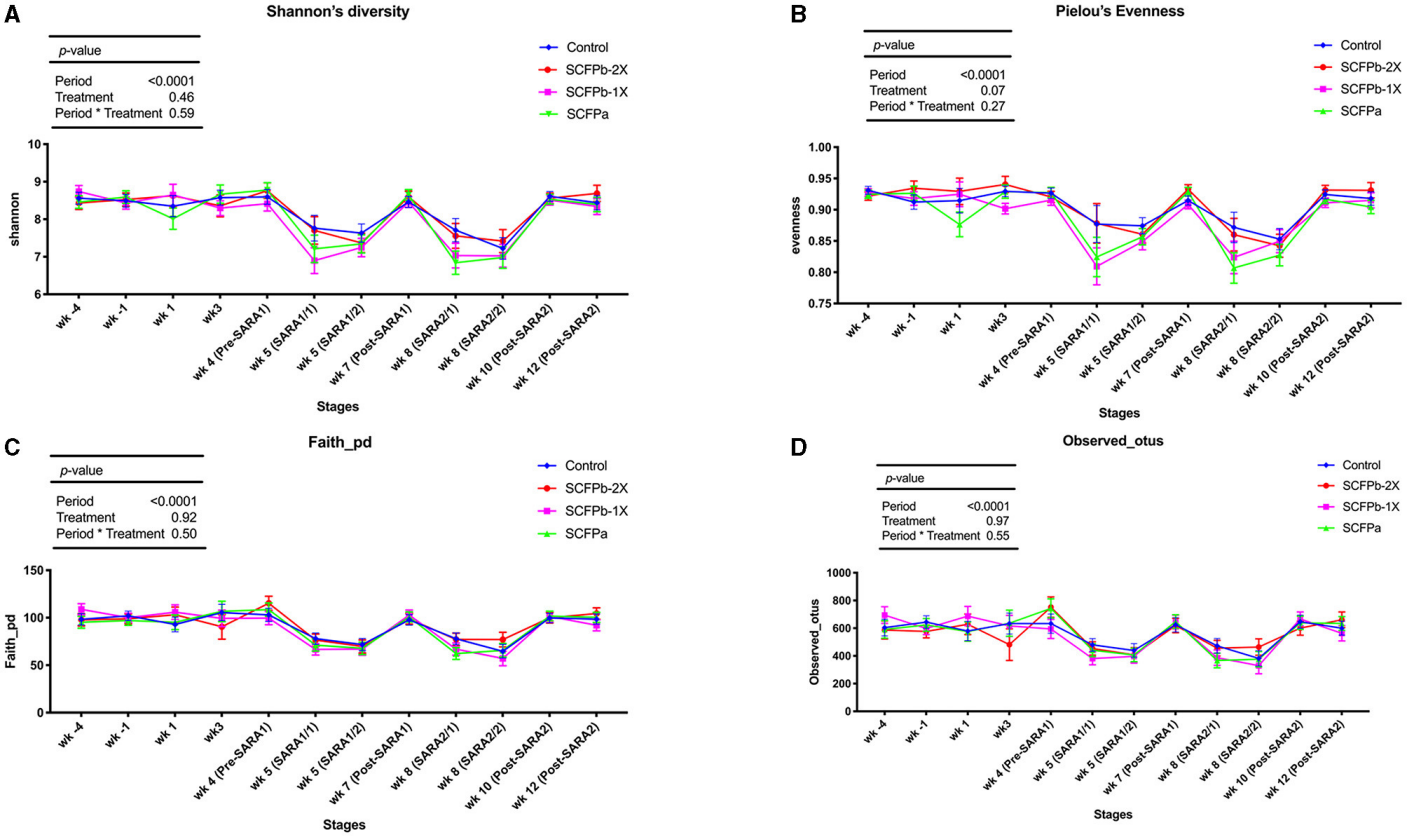
Alpha-diversity dynamics of rumen solids microbiota. Dynamics of **(A)** Shannon diversity index, **(B)** Pielou's evenness index, **(C)** Faith's phylogenetic diversity, and **(D)** Observed features within treatments (control, SCFPa, SCFPb-1X, and SCFPb-2X) from 4 weeks before until 12 weeks after calving. SARA challenges were conducted on week 5 and week 8 after parturition. Rumen samples were taken weekly but two times during SARA weeks (SARA1/1, SARA1/2, SARA2/1, SARA2/2). Week 4 was considered as pre-SARA1, week 7 as post-SARA1, and weeks 10 and 12 as post-SARA2.

**Figure 2:**
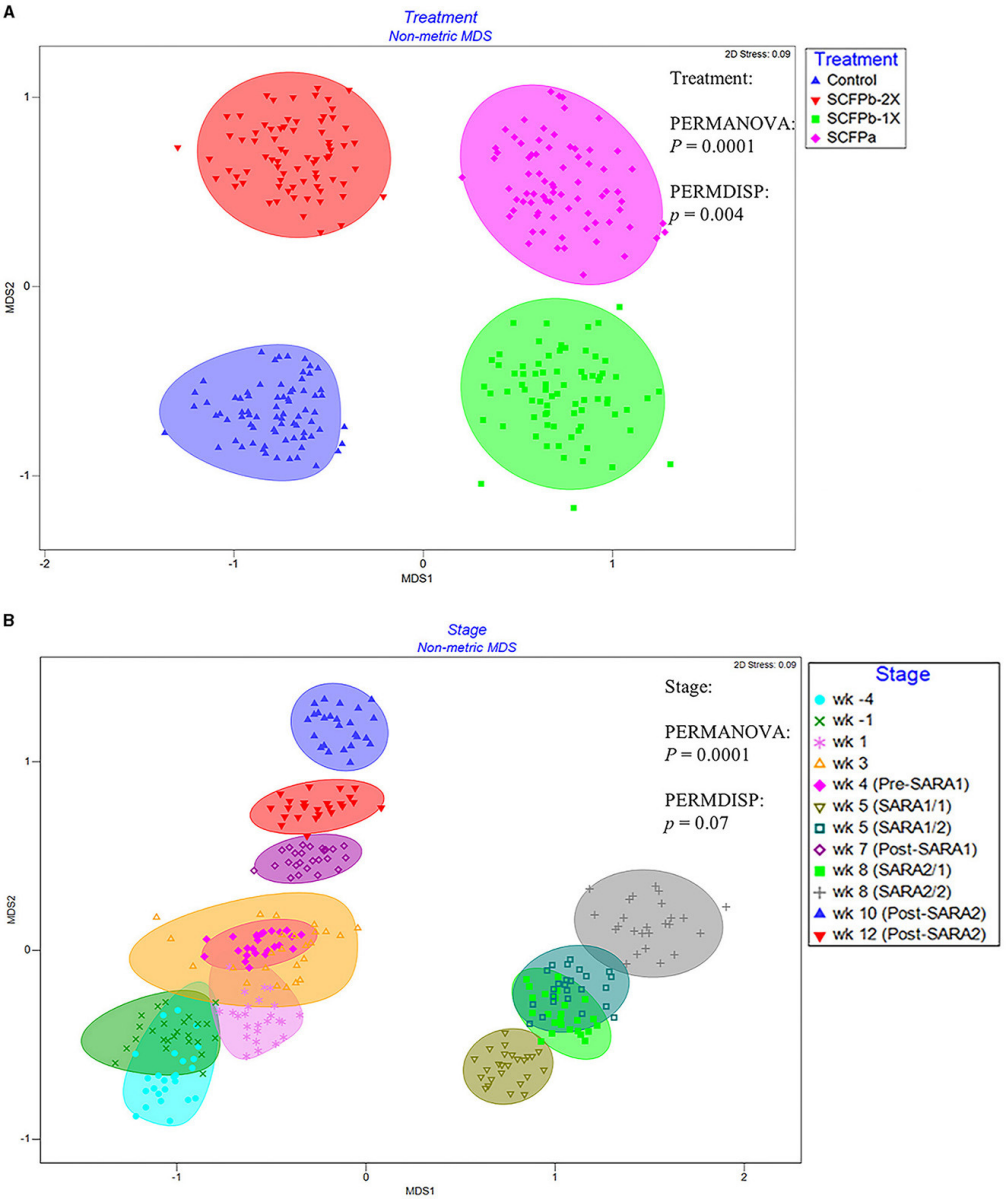
nMDS of Bray-Curtis distances of rumen solids microbial communities among treatments **(A)** and stages **(B)**. The ASV table was normalized using cumulative sum scaling (CSS) transformation. Permutational multivariate analysis of variance (PERMANOVA) was used to detect the distinction of clustering patterns between treatments and stages. PERMDISP analysis in Primer 7 was used to detect the effect of the dispersions on the β-diversity of the rumen solids microbiota. *p* < 0.05 was considered a significant difference. The effect of the block was considered as a random factor in all comparisons. The experimental stage was started 4 weeks before and until 12 weeks after calving. SARA challenges were conducted on week 5 and week 8 after parturition. Rumen samples were taken weekly but two times during SARA weeks (SARA1/1, SARA1/2, SARA2/1, SARA2/2). Week 4 was considered as Pre-SARA1, week 7 as Post-SARA1, and weeks 10 and 12 as Post-SARA2.

A total of 22,622 ASVs were found in samples with an average of 19,738 ± 6,072 reads per sample through 16S rRNA gene sequencing. The SARA challenges reduced the richness and diversity of microbiota in rumen solids (*p* < 0.001, Supplementary Table 1, Figure 1). There was no interaction effect of treatment and stage on alpha-diversity indices (*p* > 0.1). Treatments tended to affect evenness indices of the rumen solids microbiota (*p* = 0.07). The SCFPa treatment had lower evenness than the control treatment (*p* = 0.05), whereas no differences were observed between the control and other SCFP treatments across experimental stages. No treatment effect was observed on Shannon's diversity, Faith's PD, or the observed features of the rumen solids microbiota (*p* > 0.1). The PERMANOVA analysis showed that SCFP (*p* = 0.0001, Figure 2A) and SARA challenges (*p* = 0.0001, Figure 2B) affected the β-diversity of rumen solids microbiota. However, the PERMDISP analysis showed a significant treatment effect (*p* = 0.004, Figure 2A) but not a significant SARA challenge effect (*p* = 0.07, Figure 2B), confirming homogeneity of dispersions among SARA stages but not treatments. The composition of the rumen solids microbiota differed among treatment groups across the SARA stages.

### 3.2 Compositional dynamics of rumen solids microbiota

In total, 14 phyla and 445 genera were identified in the microbiota of rumen solids. The most abundant bacterial phyla in rumen solids were Firmicutes (77–80%), Bacteroidetes (16–19%), Actinobacteria (0.8–1.1%), Proteobacteria (0.5–0.8%), and Fibrobacteres (0.1–0.3%). The average relative abundances of these phyla during the non-SARA and SARA stages in each treatment group are summarized in Table 1. There was a treatment effect on the relative abundance of Fibrobacteres across experimental stages (*p* < 0.01), and the relative abundance of this phylum was lower in the SCFPb-1X treatment compared with the control and SCFPb-2X treatments (*p* < 0.01). No treatment effect was observed on the relative abundances of other phyla (*p* > 0.05). The effect of the SARA stage was significant for all phyla (*p* < 0.05). There were no interaction effects of treatment and SARA stage with the relative abundance of any identified phyla (*p* > 0.1). The relative abundances of Actinobacteria were higher in the SARA2 stage than in the post-SARA2 stage (1.15 vs. 0.84%, *p* < 0.05). During the SARA2 stage, the relative abundance of Proteobacteria was higher than the non-SARA stages (*p* < 0.05), while SARA1 tended to increase more than post-SARA2 (0.71 vs. 0.46%, *p* = 0.07). Meanwhile, both grain-based SARA challenges reduced the relative abundance of Fibrobacteres (*p* < 0.001). Neither the SARA stages nor the SCFP treatment affected the relative abundances of Firmicutes and Bacteroidetes. There was also no treatment or SARA stage effect on the ratio of Firmicutes-to-Bacteroidetes (F:B, *p* > 0.1).

**Table 1:** Effects of treatment (Control, SCFPa, SCFPb-1X, and SCFPb-2X), stage of subacute ruminal acidosis (SARA) induction (Pre-SARA1, SARA1, Post-SARA1, SARA2, and Post-SARA2) on the relative abundances (%) of the main phyla within the rumen solids microbiota in lactating dairy cows.

**Item**	**Treatment** ^**1**^				**Stage** ^**2**^					**SEM**	***p*** **-values** ^**3**^					
	**Control**	**SCFPa**	**SCFPb-1X**	**SCFPb-2X**	**Pre- SARA1**	**SARA1**	**Post- SARA1 (week 7)**	**SARA2**	**Post- SARA2 (week 10)**		**Treat**	**Stage**	**Treat × Stage**	**Contrasts** ^**4**^		
														**1**	**2**	**3**
Actinobacteria	0.93	1.06	1.02	0.85	0.90^ab^	1.09^ab^	0.86^ab^	1.15^a^	0.84^b^	0.08	0.33	0.04	0.11	0.65	0.23	0.17
Bacteroidetes	17.26	16.56	16.82	18.39	15.79	18.60	17.22	18.23	16.44	0.86	0.53	0.21	0.48	0.99	0.34	0.23
Firmicutes	78.78	79.39	79.21	77.91	80.28	77.72	78.92	77.39	79.82	0.96	0.73	0.21	0.55	0.96	0.49	0.36
Proteobacteria	0.54	0.59	0.72	0.54	0.49^b^	0.71^abA^	0.51^b^	0.89^a^	0.46^bB^	0.07	0.31	< 0.001	0.83	0.40	0.70	0.10
Fibrobacteres	0.26^a^	0.23^ab^	0.16^b^	0.27^a^	0.44^a^	0.10^b^	0.31^a^	0.09^b^	0.38^a^	0.04	0.006	< 0.001	0.53	< 0.001	0.36	0.01
F:B^5^	4.59	4.87	4.75	4.25	5.11	4.26	4.59	4.28	4.87	0.28	0.53	0.33	0.52	0.60	0.24	0.42

^a, b^Means in a row with different superscripts among treatments or stages are different (*p* < 0.05).

^A, B^Means in a row with different superscripts among treatments tended to be different (0.05 ≤ *p* < 0.1).

^1^Treatment: Control = 140 g/d ground corn; SCFPa = 14 g/d Diamond V Original XPC mixed with 126g/d ground corn; SCFPb-1X = 19 g/d NutriTek mixed with 121 g/d ground corn; SCFPb-2X = 38 g/d NutriTek mixed with 102 g/d ground corn.

^2^Stage: SARA was induced during weeks 5 (SARA1) and 8 (SARA2) post-calving. Week 4 was considered as Pre-SARA1, week 7 as Post-SARA1, and week 10 as Post-SARA2.

^3^Statistical analyses were conducted on log-transformed data for Actinobacteria and Proteobacteria, original data for Bacteroidetes and Firmicutes, and Box-cox data for Fibrobacteres. Presented means are original values prior to transformation.

^4^Contrasts: 1 = Control vs. SCFP, 2 = SCFPa vs. SCFPb, 3 = SCFPb-1X vs. SCFPb-2X.

^5^F:B: Ratio of Firmicutes to Bacteroidetes.

Comparisons of compositional shifts in proportions of Firmicutes and Bacteroidetes between the control and SCFP treatment groups are shown in Supplementary Figure 1. No significant differences in the fitting splines of Firmicutes and Bacteroidetes were observed between the control and SCFP treatments during the SARA challenges.

**Figure 3:**
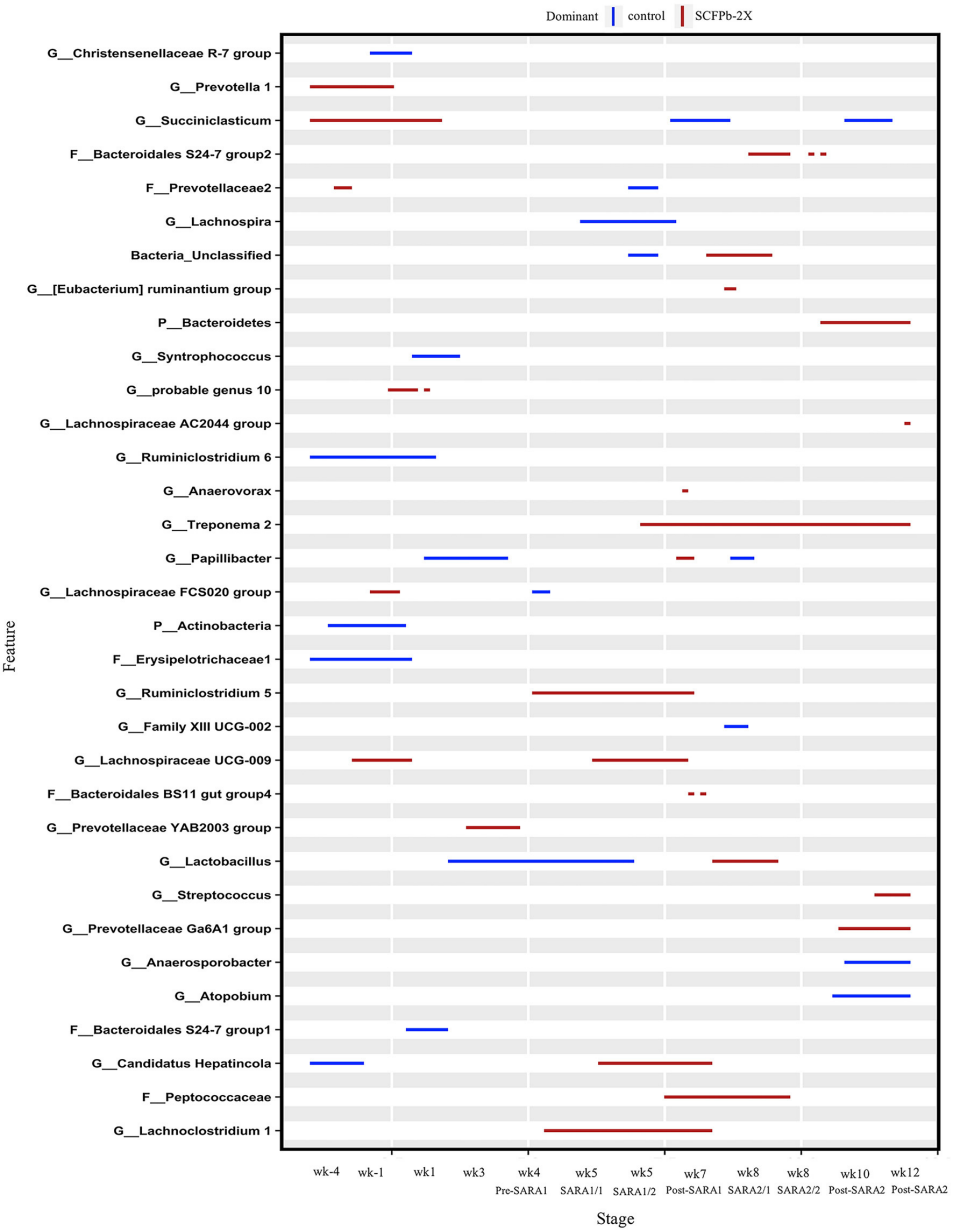
Differentially abundant taxa in rumen solids microbiota in control vs. SCFPb-2X. The graph summarizes data that were generated using the Metagenomic Longitudinal Differential Abundance (MetaLonDA) method. The significant time intervals were identified when *p* < 0.05 after multiple testing corrections using the Benjamini-Hochberg False Discovery Rate. X-axes represent the stage relative to parturition. Y-axes represent taxa that were promoted by control (blue lines) or SCFPb-2X groups (red lines) during corresponding stages.

**Figure 4:**
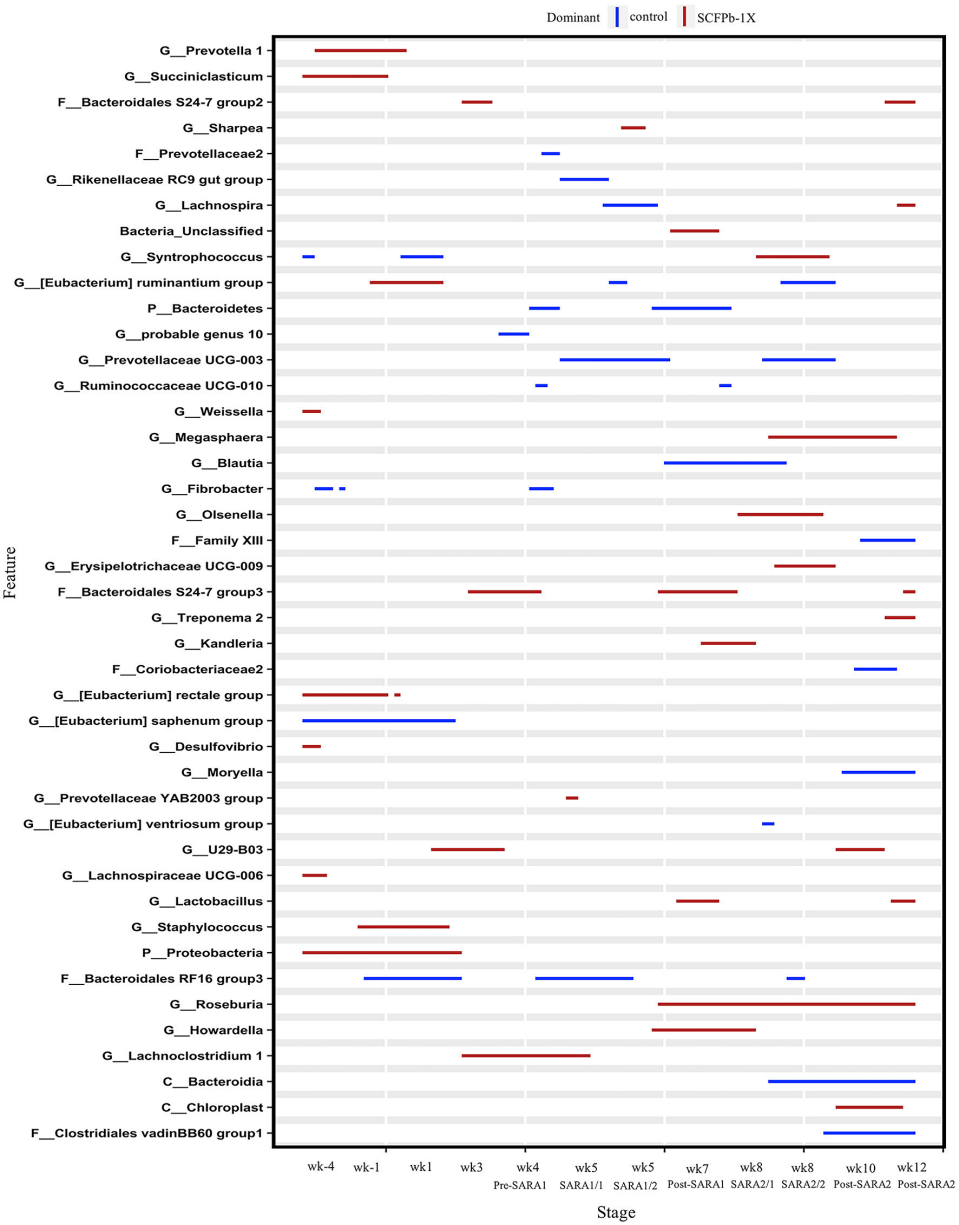
Differentially abundant taxa in rumen solids microbiota in control vs. SCFPb-1X. The graph summarizes data that were generated using the Metagenomic Longitudinal Differential Abundance (MetaLonDA) method. The significant time intervals were identified when *p* < 0.05 after multiple testing corrections using the Benjamini-Hochberg False Discovery Rate. X-axes represent the stage relative to parturition. Y-axes represent taxa that were promoted by control (blue lines) or SCFPb-1X groups (red lines) during corresponding stages.

**Figure 5:**
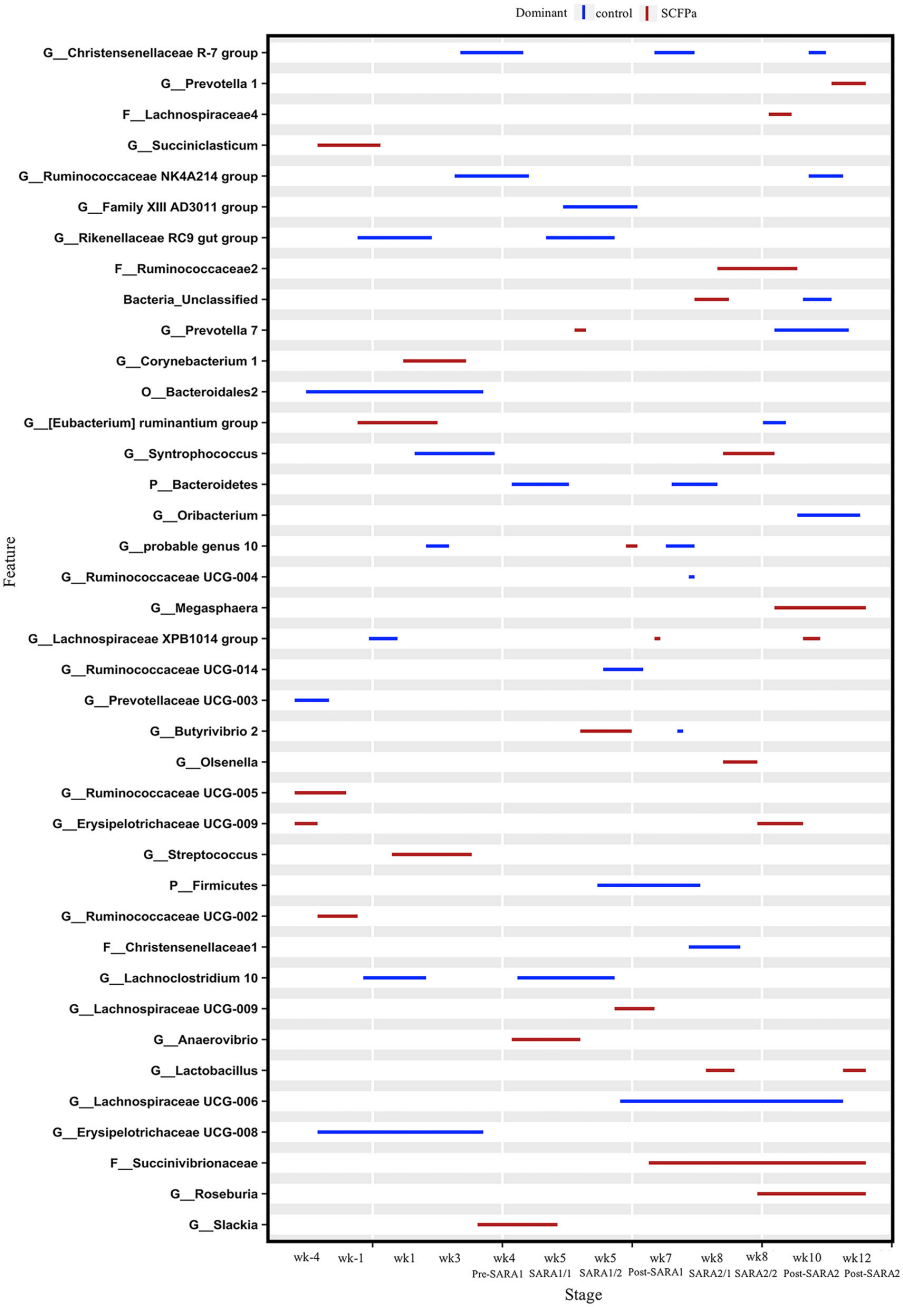
Differentially abundant taxa in rumen solids microbiota in control vs. SCFPa. The graph summarizes data that were generated using the Metagenomic Longitudinal Differential Abundance (MetaLonDA) method. The significant time intervals were identified when *p* < 0.05 after multiple testing corrections using the Benjamini-Hochberg False Discovery Rate. X-axes represent the stage relative to parturition. Y-axes represent taxa that were promoted by control (blue lines) or SCFPa groups (red lines) during corresponding stages.

At the genus level, taxa that were significantly more abundant within each stage are shown in Figures 3–5. The relative abundances of 33 taxa were increased by either control or SCFPb-2X treatments across the 16-week experimental period (Figure 3). Briefly, the SCFPb-2X treatment increased the relative abundances of several genera, including members of *Ruminiclostridium, Lachanospiraceae UCG-009, Candidatus Hepatincola*, and *Lachnoclostridium* during the first SARA challenge (SARA1). The SCFPb-2X treatment also increased members of the family Bacteroidales S24-7 group, *Treponema, Lactobacillus*, and family Peptococcaceae during SARA2 (*p* < 0.05). When comparing the control and SCFPb-1X treatments, the relative abundances of 43 bacterial taxa were increased by one of these two treatment groups within each SARA stage (Figure 4). Briefly, the SCFPb-1X treatment increased the relative abundance of *Sharpea* during the first SARA challenge (SARA1), as well as members of *Erysipelotrichaceae UCG-009, Megasphaera*, and Lachospiraceae (*Syntrophococcus*) compared to the control during SARA2 (*p* < 0.05).

A comparison of the control and SCFPa treatments revealed that the relative abundances of 39 bacterial taxa were increased by one of these two treatments (Figure 5). Briefly, the SCFPa treatment increased the relative abundances of *Prevotella, Butyrivibrio*, and *Anaerovibrio* during the SARA1 stage. It increased these abundances of members of Ruminococcaceae, Succinivibrionaceae, *Syntrophococcus, Olsenella*, and *Lactobacillus* during the SARA2 stage, as well as *Corynebacterium, [Eubacterium] ruminantium and Streptococcus* during the first week after calving (*p* < 0.05).

### 3.3 Predicted functionality of rumen solids microbiota

The predicted functionality of rumen solids microbiota revealed 254 endogenous third-level KEGG pathways using CowPi. Of these, 150 were considered rumen microbial metabolic pathways. The comparison between non-SARA and SARA stages in each treatment group is shown in Supplementary Figures 2–5. Five pathways related to carbohydrate metabolism, including butanoate metabolism, propanoate metabolism, pyruvate metabolism, carbohydrate digestion and absorption, and starch and sucrose metabolism, were inhibited by the SARA challenges in all treatment groups. These challenges inhibited four amino acid metabolic pathways, including alanine, aspartate, and glutamate metabolism; lysine biosynthesis, cysteine, and methionine metabolism; and valine, leucine, and isoleucine biosynthesis in all treatment groups. However, these challenges only inhibited histidine metabolism in the control and SCFPa treatment groups. Additionally, SARA promoted the synthesis and degradation of ketone bodies in the control and SCFPb-1X treatment groups but not in the SCFPa and SCFPb-2X groups.

**Figure 6:**
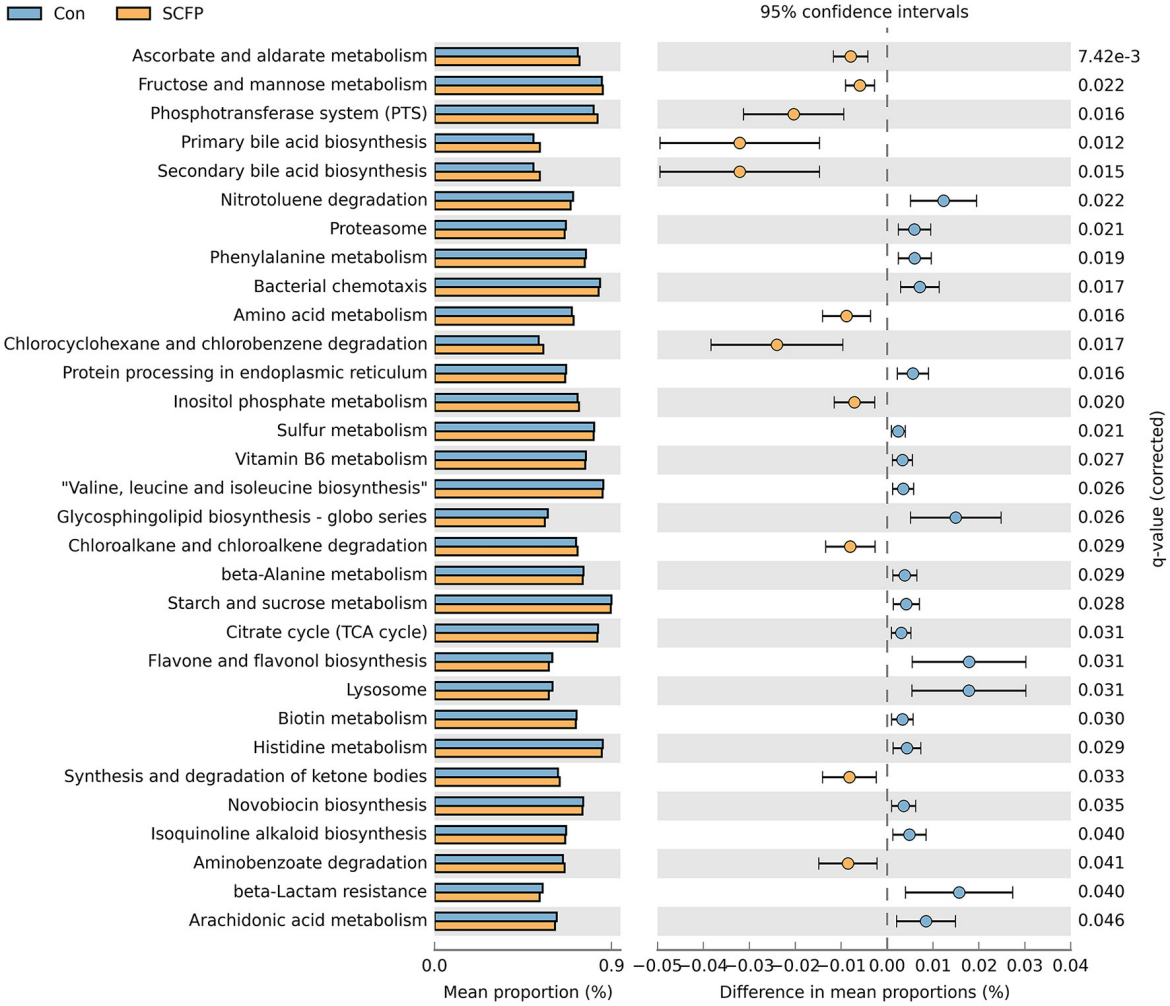
Differences in predicted functions of rumen solids microbiota between control and SCFP groups during SARA. Functionalities of rumen solids microbiota were predicted using CowPi. Output was analyzed using STAMP following log_transformation and False Discovery Rate correction. Significant differences were considered as *p* < 0.05.

The comparison between the control and SCFP groups during the SARA challenges is shown in Figure 6. Compared with control, SCFP supplementation promoted three carbohydrate metabolic pathways, including ascorbate and aldarate metabolism, fructose and mannose metabolism, and inositol phosphate metabolism. It inhibited two carbohydrate metabolic pathways, including starch and sucrose metabolism and citrate cycle (TCA cycle) during SARA challenges. Furthermore, SCFP supplementation inhibited three amino acid metabolic pathways, including valine, leucine, and isoleucine biosynthesis; histidine metabolism; and phenylalanine metabolism. It promoted one amino acid metabolic pathway (amino acid metabolism) compared with the control treatment during SARA challenges. In addition, SCFP supplementation promoted three lipid metabolic pathways, including primary bile acid biosynthesis, secondary bile acid biosynthesis, and synthesis and degradation of ketone bodies, but inhibited one lipid metabolic pathway (arachidonic acid metabolism) compared with the control treatment during the SARA challenges. A comparison of predicted microbial functionalities between the control treatment and each SCFP treatment group during SARA showed that three carbohydrate pathways (including ascorbate and aldarate metabolism, fructose and mannose metabolism, and inositol phosphate metabolism), one amino acid metabolism, and three lipid metabolic pathways (including primary bile acid biosynthesis, secondary bile acid biosynthesis, and synthesis and degradation of ketone bodies) were promoted by the SCFPa treatment (*p* < 0.05, Supplementary Figure 6). However, no differences in these predicted functionalities were observed between the control and SCFPb treatment groups.

### 3.4 Effect of SCFP supplementation and SARA inductions on co-occurrence patterns of rumen solids microbiota

**Figure 7:**
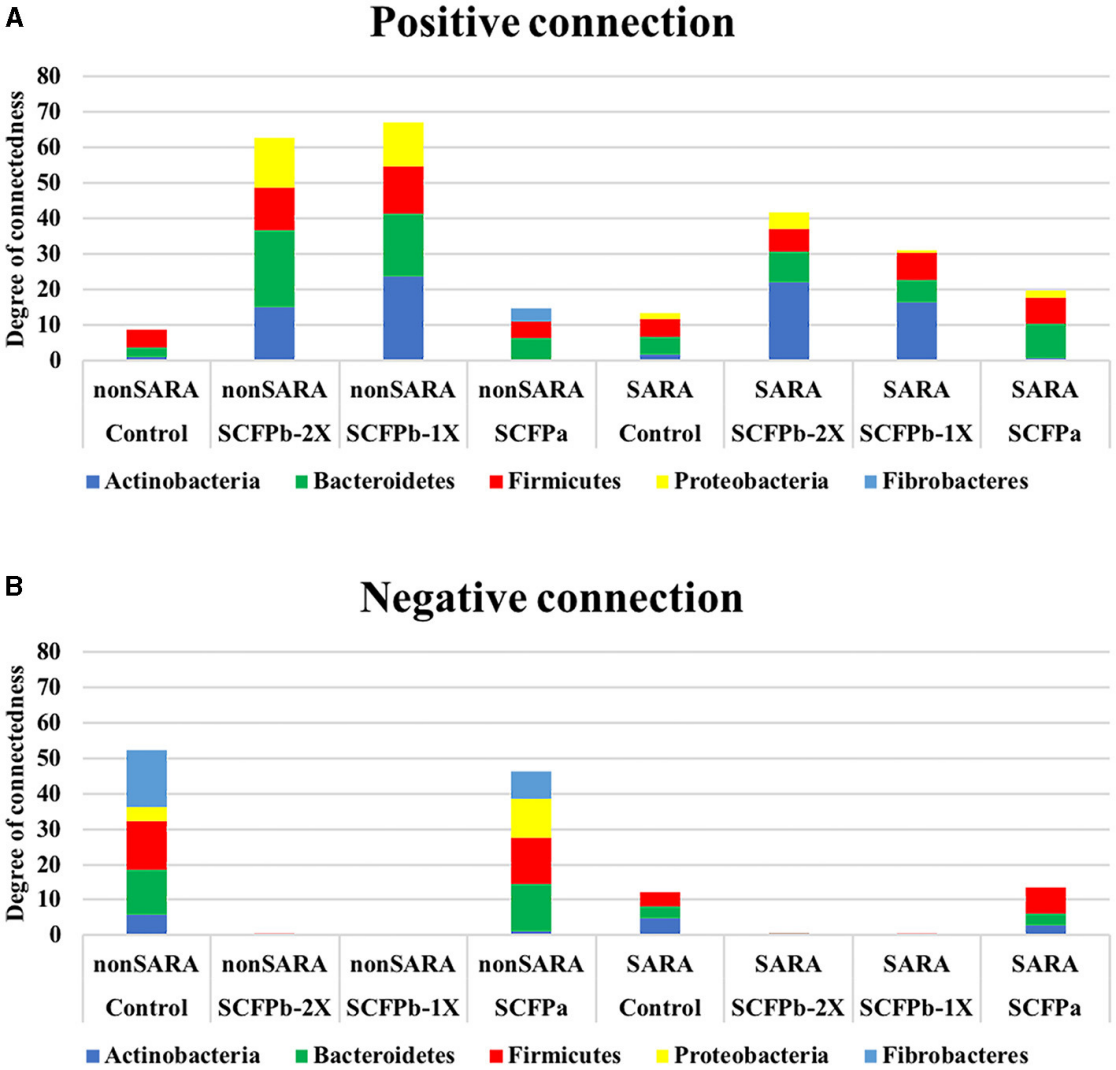
Microbial interaction networks. The degree of connections for each phylum was normalized by dividing the total number of positive and negative edges observed for each phylum by their relative abundance in the community. **(A)** Normalized positive connections for the dominant bacteria phyla within each treatment group during non-SARA (Pre-SARA1, Post-SARA1, and Post-SARA2) and SARA (SARA1/1, SARA1/2, SARA2/1, SARA2/2) stages. **(B)** Normalized negative connections for the dominant bacteria phyla with each treatment group during non-SARA and SARA stages.

The SARA challenges reduced the total number of significant associations among bacterial taxa in rumen solids microbiota (Figure 7A). No negative connections were detected in the SCFPb treatment group during the SARA challenges (Figure 7B). The relative degree of connectedness of each phylum (total number of positive and negative edges observed for each phylum divided by its relative abundance in the community) varied among treatment groups during the SARA stages.

During non-SARA stages, the degrees of positive connections were higher in SCFP treatment groups compared to the control treatment group, and these connections were higher in the SCFPb treatment group than in the SCFPa treatment group (Figure 7A). Bacteroidetes had the highest degree of positive connections in the SCFP treatment groups. In descending order, Bacteroidetes, Actinobacteria, and Proteobacteria showed high degrees of positive connections in the SCFPb treatment groups, whereas Firmicutes and Fibrobacteres showed high degrees of positive connections in the SCFPa treatment group. Meanwhile, Firmicutes had the highest degree of connections in the control treatment group.

Across SARA challenges, the SCFP treatment groups had higher positive connections than the control treatment group, and these connections were higher in the SCFPb treatment group than in the SCFPa treatment group (Figure 7A). Actinobacteria contributed the most to the positive connections in SCFPb treatment groups, followed by Bacteroidetes and Firmicutes. In the SCFPa treatment group, Bacteroidetes had the highest number of positive connections. Bacteroidetes and Firmicutes contributed equally to positive connections in the control treatment group (Figure 7A). Actinobacteria, Bacteroidetes, and Firmicutes had equal numbers of negative connections in the control group, whereas in the SCFPa group, Firmicutes was the major contributor to the negative connections, followed by Bacteroidetes and Actinobacteria (Figure 7B).

**Figure 8:**
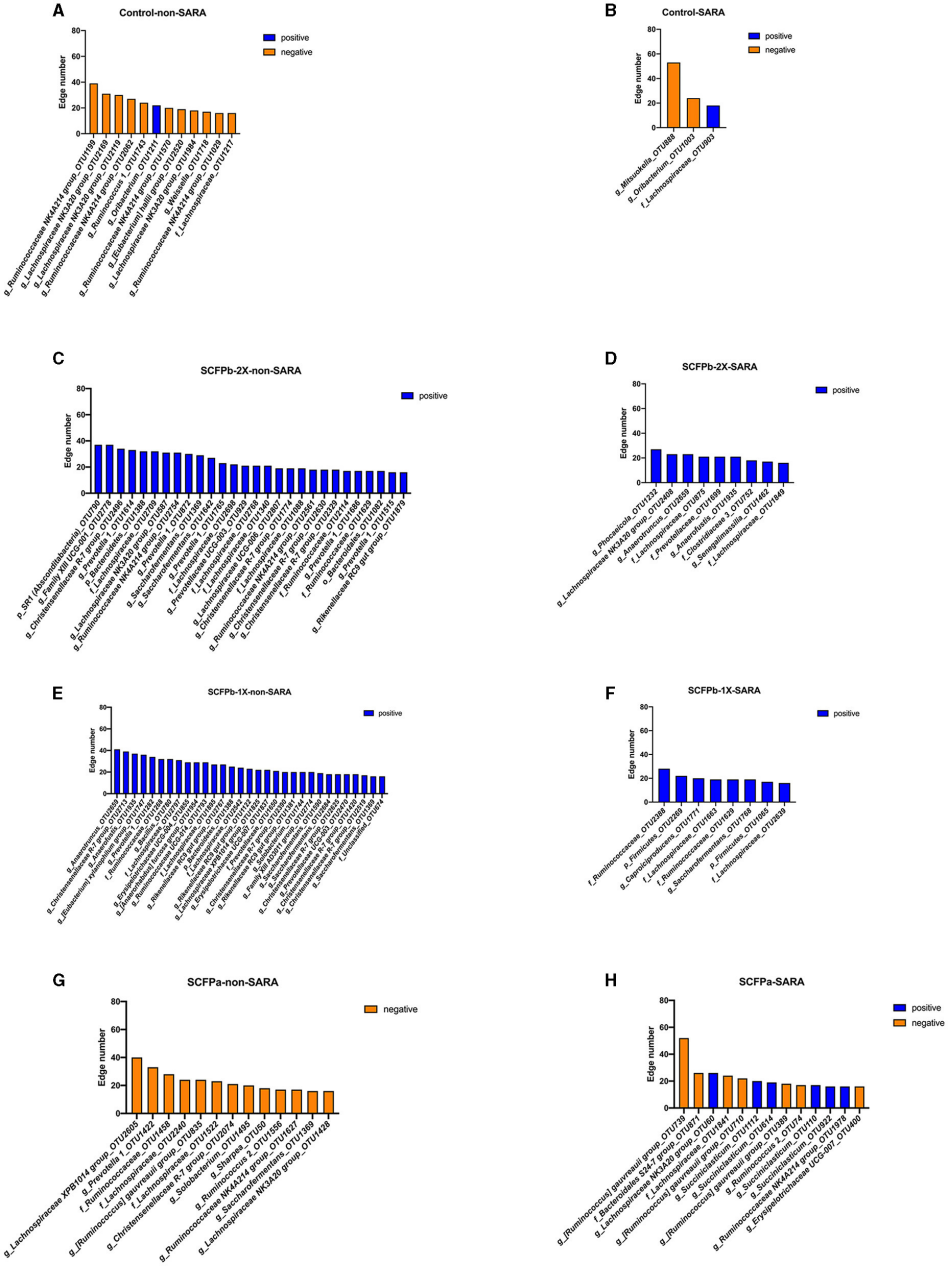
Distribution of hub taxa within each treatment group during non-SARA and SARA stages. Hub taxa were identified as OTUs with more than 15 connections with other members of rumen solids microbiota within each treatment group during non-SARA (Pre-SARA1, Post-SARA1, and Post-SARA2) and SARA (SARA1/1, SARA1/2, SARA2/1, SARA2/2) stages**. (A, B)** control; **(C, D)** SCFPb-2X; **(E, F)** SCFPb-1X; **(G, H)** SCFPa. Orange color represents negative connections while blue color represents positive connections.

Hub taxa were identified as taxa with a high number (>15) of positive or negative connections with other members of the community (Figure 8). The SARA challenges reduced the number of hub taxa in the control and SCFPb treatment groups but not in the SCFPa treatment group. In the control treatment group, all hub taxa were from the Firmicutes phylum during both SARA and non-SARA stages. During non-SARA stages, cows in the control group had nine negatively connected hub taxa (three from the *Lachnospiraceae NK3A20 group*, one from the family Lachnospiraceae*, four* from the *Ruminococcaceae NK4A214 group*, and one from *Ruminococcus*) and one positively connected hub taxon (from genera *Oribacterium*) (Figure 8A). During the SARA stages, control cows had two negatively connected hub taxa (one from *Oribacterium* and one from *Mitsuokella*) and one positively connected taxon from the family Lachnospiraceae (Figure 8B).

In the SCFPb-2X treatment group, all positively connected hub taxa were from the Bacteroidetes and Firmicutes phylum during non-SARA stages and from the Bacteroidetes, Firmicutes, and Actinobacteria phyla during SARA stages. During non-SARA stages, SCFPb-2X cows had positively connected hub taxa from Prevotellaceae (one from *Prevotellaceae UCG-003*, five from *Prevotella*), Christensenellaceae (four from genera *Christensenellaceae R-7 group*), Lachnospiraceae (one from *Lachnospiraceae NK3A20 group*, five from family Lachnospiraceae, one from *Lachnospiraceae UCG-006*), Ruminococcaceae (two from *Ruminococcaceae NK4A214 group*, two from family Ruminococcaceae and two from *Saccharofermentans*) and one from genera *Rikenellaceae RC9 gut group* (Figure 8C). During SARA stages, SCFPb-2X cows had positively connected hub taxa from Lachnospiraceae (one from genera *Lachnospiraceae NK3A20 group* and two from family Lachnospiraceae), Ruminococcaceae (one from genera *Anaerotruncus*), one from family Prevotellaceae, family Bacteroidales Incertae Sedis (one from genara *Phocaeicola*) and family Coriobacteriaceae (one from genera *Senegalimassilia*) (Figure 8D).

Similarly, all positively connected hub taxa in the SCFPb-1X treatment group were from the Bacteroidetes and Firmicutes phyla during non-SARA stags and from the Firmicutes phylum during SARA stages. During the SARA stages, hub taxa were from Ruminococcaceae (one from the genera *Anaerotruncus*, three from the genera *Saccharofermentans*, one from family Ruminococcaceae, and one from *Ruminococcaceae UCG-014*), Christensenellaceae (five from *Christensenellaceae R-7 group*), Lachnospiraceae (one from *[Eubacterium] xylanophilum group*, one from *Lachnospiraceae XPB1014 group*, and three from family Lachnospiraceae), Erysipelotrichaceae (one from *Erysipelotrichaceae UCG-004*, one from *Erysipelotrichaceae UCG-007*, one from *Solobaterium*, one from *[Anaerorhabdus] furcosa group*), Bacillaceae (one from *Bacillus*), Eubacteriaceae (one from *Anaerofustis*), *Rikenellaceae* (three from *Rikenellaceae RC9 gut group*), Prevotellaceae (one from *Prevotella*, one from Prevotellaceae *UCG-003*, one from family Prevotellaceae) and one from Family XIII AD3011 group (Figure 8E). During SARA stages, hub taxa were from Ruminococcaceae (one from *Caproiciproducens*, one from *Saccharofermentans*, two from family Ruminococcaceae) and two from family Lachnospiraceae (Figure 8F).

In the SCFPa treatment group, hub taxa that were negatively connected were from Bacteroidetes and Firmicutes during non-SARA stages and had a combination of positively and negatively connected hub taxa from Firmicutes during SARA stages. During non-SARA stages, SCFPa cows had hub taxa from Lachnospiraceae (one from *Lachnospiraceae XPB1014 group*, one from *[Ruminococcus] gauvreauii group*, one from *Lachnospiraceae NK3A20 group* and two from family Lachnospiraceae), Ruminococcaceae (one from *Ruminococcus*, one from *Ruminococcaceae NK4A214 group*, one from *Saccharofermentans* and one from family Ruminococcaceae), Erysipelotrichaceae (one from *Solobacterium* and one from *Sharpea*), *Christensenellaceae* (one from *Christensenellaceae R-7 group*), and Prevotellaceae (one from *Prevotella*) (Figure 8G). During SARA challenges, cows had six positively connected hub taxa (one from *Lachnospiraceae NK3A20 group*, four from genera *Succiniclasticum*, and one from *Ruminococcaceae NK4A214 group*) and seven negatively connected taxa (three from *[Ruminococcus] gauvreauii group*, one from family Lachnospiraceae, one from family Bacteroidales S24-7 group, one from *Erysipelotrichaceae UCG-007*, and one from *Ruminococcus*) (Figure 8H).

As shown in Table 2, results from the NetCoMi analysis indicated that network characteristics, including modularity (*p* = 0.04), clustering coefficient (*p* = 0.09), positive edge percentage (*p* = 0.07), and edge density were increased during SARA stages compared to non-SARA stages in the control group. In the SCFPb-1X treatment group, the clustering coefficient (*p* = 0.02), positive edge percentage (*p* < 0.001), edge density (*p* < 0.001), and natural connectivity (*p* < 0.001) were higher during SARA stages compared to non-SARA stages. In the SCFPa treatment group, the clustering coefficient (*p* = 0.02), positive edge percentage (*p* < 0.001), edge density (*p* < 0.001), and natural connectivity (p < 0.001) were also increased, while modularity decreased (*p* = 0.04) during SARA stages compared to non-SARA stages. However, there was no difference in network characteristics between non-SARA and SARA stages in the SCFPb-2X treatment group.

**Table 2:** Comparison of network properties in each treatment group (control, SCFPa, SCFPb-1X, and SCFPb-2X) between non-SARA and SARA stages.

**Treatment^1^**	**Characteristics**	**Stage** ^**2**^		**Absolute difference**	***p*-value^3^**
		**Non-SARA**	**SARA**		
Control	Number of components	29	9	20	0.006
	Clustering coefficient	0.35	0.48	0.13	0.09
	Modularity	0.45	0.59	0.14	0.04
	Positive edge percentage	78.76	94.04	15.27	0.07
	Edge density	0.03	0.05	0.01	0.09
	Natural connectivity	0.01	0.02	0.006	0.16
SCFPa	Number of components	24	4	20	0.002
	Clustering coefficient	0.30	0.51	0.21	0.007
	Modularity	0.61	0.46	0.15	0.04
	Positive edge percentage	82.52	100	17.47	< 0.001
	Edge density	0.03	0.12	0.09	< 0.001
	Natural connectivity	0.02	0.07	0.05	< 0.001
SCFPb-1X	Number of components	23	7	16	0.002
	Clustering coefficient	0.43	0.63	0.2	0.02
	Modularity	0.30	0.22	0.07	0.42
	Positive edge percentage	70.58	99.36	28.77	< 0.001
	Edge density	0.05	0.17	0.11	< 0.001
	Natural connectivity	0.03	0.14	0.11	< 0.001
SCFPb-2X	Number of components	19	15	4	0.58
	Clustering coefficient	0.44	0.44	0	0.99
	Modularity	0.44	0.36	0.07	0.36
	Positive edge percentage	81.77	75.60	6.17	0.39
	Edge density	0.04	0.05	0.01	0.21
	Natural connectivity	0.03	0.03	0.001	0.73

^1^Treatment: control = 140 g/d ground corn; SCFPa = 14 g/d Diamond V Original XPC mixed with 126 g/d ground corn; SCFPb-1X = 19 g/d NutriTek mixed with 121 g/d ground corn; SCFPb-2X = 38 g/d NutriTek mixed with 102 g/d ground corn.

^2^Stage: SARA was induced during week 5 (SARA1) and week 8 (SARA2) after parturition. The non-SARA stage includes pre-SARA1 (week 4), post-SARA1 (week 7), and post-SARA2 (weeks 10 and 12). SARA stage includes SARA1 and SARA2.

^3^*p*-value: Significance was considered as *p* < 0.05, and a tendency was considered as 0.05 ≤ *p* < 0.1.

### 3.5 Correlation between hub taxa, biodiversity metrics of rumen solids microbiota, and rumen fermentation characteristics

**Figure 9:**
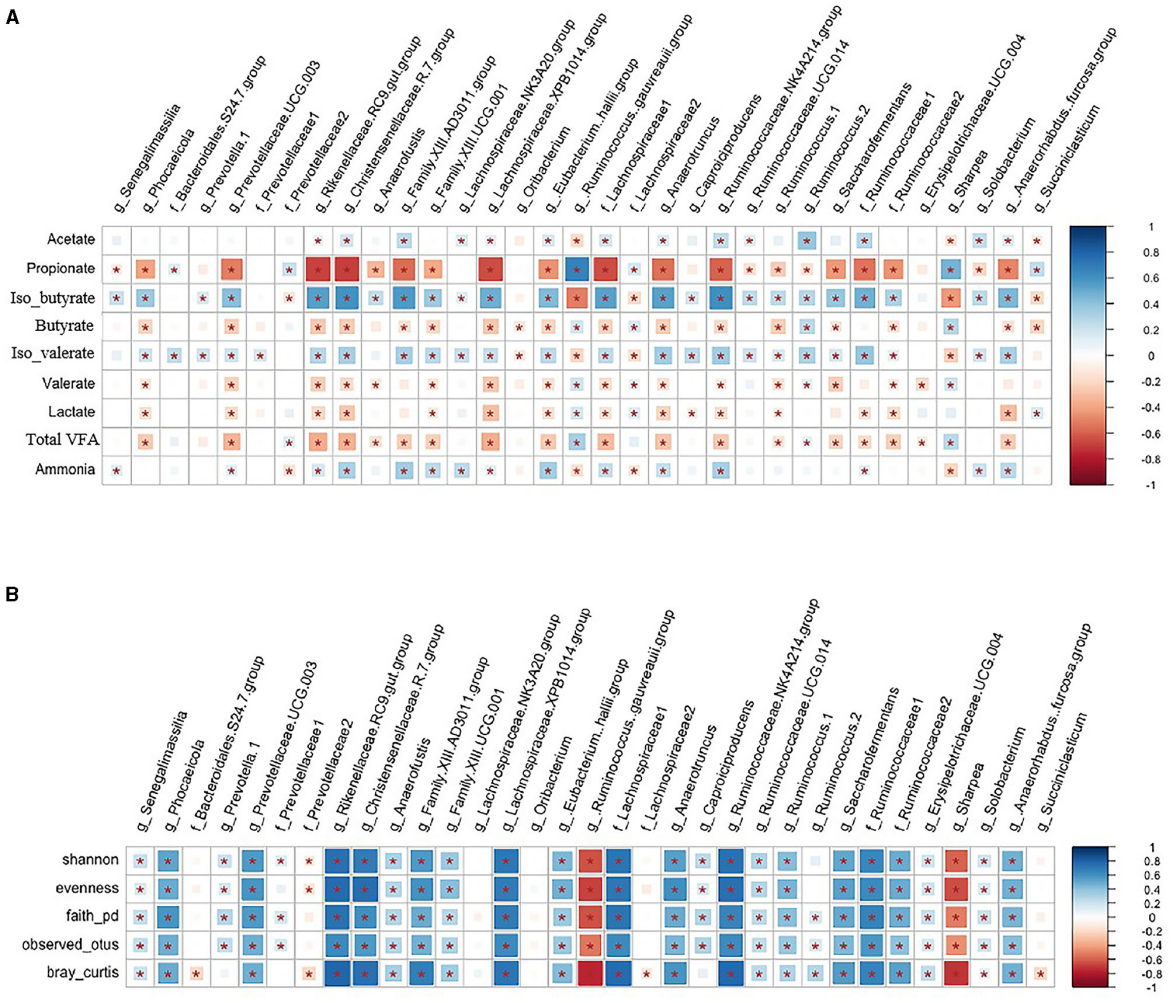
**(A, B)** Correlation among hub taxa and rumen fermentation characteristics and biodiversity metrics of rumen solids microbiota. Spearman's correlation coefficient was used to explore the relationships between the relative abundances of rumen solids hub taxa and community α-diversity (Shannon, Evenness, Faith_PD, and Observed_Features), β-diversity (Bray-Curtis dissimilarities) and rumen fermentation characteristics (VFAs and ammonia concentrations). *indicates *p* < 0.05. The color ramp and the size of the squares indicate the type and strength of Spearman's correlation coefficient (rho): rho = 1 shows a strong positive correlation, and rho = −1 shows a strong negative correlation between the two parameters.

The association between hub taxa and biodiversity metrics of rumen solids microbiota, rumen VFA, and ammonia concentrations that were published in a companion paper (Khalouei et al., 2020) are shown in Figure 9. A group of taxa from Bacteroidetes, including *Rikenellaceae RC9 gut group*, Firmicutes, including *Christensenellaceae R-7 group*, Family XIII AD3011 group, *Lachnospiraceae XPB1014 group*, unclassified family *Lachnospiraceae, Anaerotruncus, Ruminococcaceae NK4A214 group*, unclassified family *Ruminococcaceae, [Anaerorhabdus] furcosa group*, and genera *Saccharofermentans* were negatively correlated with rumen concentrations of propionate, butyric, valerate, and lactate (*p* < 0.05). However, they were positively correlated with rumen concentrations of acetate and ammonia and with α-, β-diversity metrics of the microbiota in solid rumen digesta (*p* < 0.05). A group of taxa from Firmicutes that included members of the *Ruminococcus gauvreauii* and *Sharpea* genera were positively correlated with rumen propionate and butyrate concentrations but were negatively correlated with rumen acetate and ammonia concentrations and with α-, β-diversity metrics of the rumen solids microbiota (*p* < 0.05).

## 4 Discussion

Postbiotics contain bioactive compounds and functional metabolites with a dual mode of action that on the one hand can prime the immune response (Vailati-Riboni et al., 2021; Guo et al., 2024) and, on the other hand can improve rumen health and fermentation by supporting the populations of hub taxa, such as fibrolytic bacteria, therefore increasing the stability and robustness of microbial community during dietary, metabolic or infectious stressors (Zhu et al., 2017; Tun et al., 2020; Ganda et al., 2023). Bacteria are the most abundant kingdom in the rumen microbial community, and rumen solids-associated bacteria have been estimated to comprise more than 70% of bacterial biomass in the rumen (Craig et al., 1987; Mullins et al., 2013). These bacteria play key roles in the degradation and fermentation of the digesta (Bickhart and Weimer, 2018). The relationship between the changes in the diet and the composition of the microbial community in the rumen liquid has been widely studied. Our study revealed the effects of two commercial postbiotics from *Saccharomyces cerevisiae* fermentation on diversity, composition, predicted functionality, and network structure of the bacterial community in rumen solids in dairy cows subjected to repeated grain-based SARA challenges.

### 4.1 Effects of grain-based SARA challenges on diversity, composition, and predicted functionality of rumen solids microbiota

Companion papers to this work showed that SARA was induced successfully, as the SARA challenges increased the duration of rumen pH below 5.6 from 8 to 186 min/d (Khalouei et al., 2020) and the concentration of rumen free-LPS from 5,012 to 63,596 endotoxin unit (EU)/mL (Guo et al., 2022), both of which are indicative of SARA (Plaizier et al., 2008, 2012). Previous studies have shown that grain-based SARA challenges reduce the rumen pH and the relative abundances of pH-sensitive microorganisms, such as fibrolytic bacteria, but increase the relative abundance of low pH tolerant and amylolytic bacteria (Cherdthong et al., 2010; Mao et al., 2013; Petri et al., 2013; Plaizier et al., 2017). These changes in fiber- and starch-degrading bacteria during SARA may further influence the rumen pH because of the lower fiber and higher starch fermentation. In our study, SARA was induced by increasing the starch content of the diet from 17.6% to 27.9% DM while decreasing the dietary NDF from 55.4% to 48.1%. Hence, we expected similar changes in the populations of rumen bacteria as in previous work.

Earlier studies reported that SARA reduces the richness and diversity of the rumen liquid microbiota (Fernando et al., 2010; Mao et al., 2013; McCann et al., 2016; Guo et al., 2024). The impact of SARA on the diversity of rumen solids microbiota has been less studied. McCann et al. (2016) reported a surprise increase in the diversity of rumen solids following 6 days of SARA induction, whereas Brede et al. (2020) observed a decrease in the diversity indices when SARA was induced in an *in vitro* RUSITEC system. In the current study, we observed a reduction in the richness and diversity of the microbiota in the solid fraction during grain-based SARA challenges. This implies that induction of SARA was able to disturb the rumen solids microbial community regardless of treatments. The PERMANOVA and PERMDISP analyses of Bray-Curtis distances also demonstrated that SARA challenges had major effects on the overall composition and beta diversity of the rumen solids microbiota.

In total, 14 phyla and 445 genera were identified in the rumen solids microbiota. The predominant phyla were Firmicutes and Bacteroidetes, which comprised more than 90% of the community. The relative abundance of Firmicutes was higher than that of Bacteroidetes in the solid fraction. It has been recognized that microbiota of rumen solids plays a major role in fiber degradation, as cellulolytic bacteria are more abundant, resulting in a greater prevalence of carbohydrate active enzymes (CAZy) in this fraction compared to the liquid fraction (Williams et al., 1989; Michalet-Doreau et al., 2001). Earlier studies have also suggested that perhaps the abundance of bacteria in the rumen solids fraction is greater than in the liquid fraction (>60% vs. < 20% of total bacteria; Yang et al., 2001); however, this claim needs to be re-examined using more accurate methods. Martin et al. (2001) reported that supplementation with barley grains decreased the rumen degradation rate of hay and the fibrolytic activities of bacteria in the rumen solids (Martin et al., 2001). Increased grain feeding is expected to increase the proportion of Firmicutes and decrease that of Bacteroidetes, at least in the rumen liquid environment (Khafipour et al., 2009b; Huo et al., 2014). McCann et al. (2016) reported that the relative abundance of Firmicutes in the rumen solids fraction decreased, and that of Bacteroidetes increased following 6 days of SARA induction. Similarly, Fernando et al. (2010) observed a gradual increase in the proportion of Bacteroidetes in the rumen when beef steers were adapted to a high-concentrate diet. In contrast with previous studies, we did not observe significant changes in relative abundances of Firmicutes and Bacteroidetes in rumen solids during grain-based SARA. This discrepancy may be due to different sampling strategies, experimental designs, and microbiome evaluation methods. We separated rumen solids and assessed the microbiota of this fraction, whereas the study of Fernando et al. (2010) evaluated the whole rumen content. Further, we used a complete randomized block design to avoid any crossover effects, whereas the above-mentioned studies incorporated a Latin square design.

The Lachnospiraceae family belongs to the phylum Firmicutes and appears to be more abundant in rumen solids fraction than in liquid fraction in cows fed in pastures (De Menezes et al., 2011). Several members of the Lachnospiraceae family have been reported to be major butyrate producers (Louis et al., 2010; Meehan and Beiko, 2014). Studies have been conducted on the correlation between the abundance of this family and feed efficiency, but the results are inconsistent (Guan et al., 2008; Myer et al., 2015; Li and Guan, 2017). These discrepancies among studies could be due to limited butyrate production by some members of this family (De Menezes et al., 2011). In our companion paper, SARA challenges increased the concentration of butyrate in the rumen liquid (Khalouei et al., 2020). Also, we observed that the abundance of members of the Lachnospiraceae family either positively or negatively correlated with the butyrate concentration in the rumen liquid environment (Guo et al., 2024). However, we found that SARA challenges did not affect the Firmicutes-to-Bacteroidetes ratio in the rumen solids or the liquid fraction.

Fibrobacteres, which are an important phylum of the cellulolytic bacteria, were reduced during SARA in this study. Consistently, Lourenco et al. (2020) reported that supplementation with grain decreases the abundance of Fibrobacteres in the rumen liquid digesta of beef calves. Fernando et al. (2010) detected a higher proportion of Fibrobacteres in rumen solids and liquid digesta in hay-fed animals than in grain-fed animals. The reduction of the relative abundance of this phylum during SARA stages was expected as the dietary starch content was higher, and that of fiber was lower in the SARA diet compared with those during non-SARA stages. Actinobacteria, a gram-positive phylum, also increased in rumen solids during SARA in our study. In this phylum, the relative abundance of the most abundant genus *Bifidobacterium* from the family Bifidobacteriaceae, a lactic acid producer, has been shown to increase during SARA challenges (Nagaraja and Titgemeyer, 2007; Plaizier et al., 2017; Monteiro and Faciola, 2020). Previous studies have also reported that the phylum Proteobacteria is more abundant in the rumen when animals are fed a high-grain diet (Fernando et al., 2010; Petri et al., 2013; Auffret et al., 2017). The relative abundance of Proteobacteria increased during SARA2 in our study, which is consistent with previous observations. The Proteobacteria phylum contains many opportunistic and pathogenic bacteria, such as *Escherichia coli*. The relative abundances of several of these pathogens can be enriched when feeding high-grain diets (Diez-Gonzalez et al., 1998; Khafipour et al., 2011; Bäumler and Sperandio, 2016; Auffret et al., 2017). Consequently, the increase in the abundance of Proteobacteria could be indicative of the dysbiosis of the rumen microbiota and that greater prevalence of opportunistic and pathogenic microorganisms may increase the risk of inflammation and metabolic dysfunction in the host (Khafipour et al., 2011; Petri et al., 2013; Shin et al., 2015).

Due to the changes in the composition of rumen solids microbiota during SARA challenges, differences in microbiome functionalities were expected. It has been reported that feeding highly fermentable carbohydrates promotes the production of propionate, butyrate, and lactate and reduces the ratio of acetate-to-propionate in the rumen (Gozho et al., 2007; Li et al., 2016; Khalouei et al., 2020). Liu et al. (2022) found that cattle fed with a grain diet had higher relative abundances of the starch-fermenting bacteria, such as Succinivibrionaceae and *Succinimonas*, and higher relative abundances of the lactate-utilizing bacteria, such as *Megasphaera* and *Acetobacter*. Our study found that the relative abundance of genera *Succiniclasticum* was positively correlated with the propionate concentration in the rumen solids digesta. As a major succinate producer, the family Succinivibrionaceae competes with methanogens for hydrogen (Pope et al., 2011). Succinate is the precursor for propionate, which is a substrate for gluconeogenesis (Yost et al., 1977; Li and Guan, 2017). Hence, a higher ratio of propionate-to-acetate may reduce methane emissions from the rumen (Russell, 1998). In our companion paper, a higher propionate-to-acetate ratio during grain-based SARA challenges was observed (Khalouei et al., 2020). Gagen et al. (2015) found acetogens in both families of Lachnospiraceae and Ruminococcaceae, which can provide hydrogen, and their abundance may increase while methane production declines. It has been reported that increases in rumen acetate can increase milk fat production as acetate is the major source of energy and substrate for milk fat synthesis in dairy cows (Urrutia and Harvatine, 2017; Urrutia et al., 2019). We determined that the relative abundances of taxa from the *Lachnospiraceae NK3A20 group, Ruminococcaceae NK4A214 group*, and *Ruminococcus* and Ruminococcaceae groups were positively related to the acetate concentration in rumen liquid and with the diversity and richness of the microbial community in the rumen solids. Therefore, the growth of those bacteria was inhibited during grain-based SARA.

Several studies have observed a positive relationship between less diversity in the rumen microbial community and feed efficiency (McCann et al., 2014; Myer et al., 2015; Li and Guan, 2017). Li and Guan (2017) showed that nitrogen metabolism activities were inhibited in efficient cows. Similarly, we found predicted nitrogen metabolism pathways such as alanine, aspartate, and glutamate metabolism; lysine biosynthesis; phenylalanine metabolism; glycine, serine, and threonine metabolism; and valine, leucine, and isoleucine biosynthesis were inhibited during SARA. Valine, leucine, and isoleucine are important contributors to microbial protein synthesis, and microbial proteins are precursors for the synthesis of milk protein in the mammary gland (Allison et al., 1966; Xue et al., 2020). Thus, the challenges of grain-based SARA in our study may reduce milk protein synthesis. That being said, our predicted functionalities of the rumen microbiome can be biased as they are based on amplicon sequencing, which can only distinguish bacteria at the genus level. Different strains from the same species can perform different functions, while several species can contribute to similar or different functions (Benson et al., 2010; Qi et al., 2024). To properly assess the functional shifts in the rumen solids microbiota during SARA induction, more comprehensive omics studies such as shotgun metagenomics and metabolomics need to be performed.

### 4.2 Effects of SCFP supplementation on diversity, composition, and predicted functionality of rumen solids microbiota

The SCFP are commonly used as rumen fermentation modifiers. It has been reported that SCFP influences rumen fermentation by promoting the growth and functionality of fibrolytic bacteria and lactate-utilizing bacteria, as well as increasing microbial protein synthesis and reducing lactate accumulation (Callaway and Martin, 1997; Zhu et al., 2017). Different from our companion study, where we showed SCFP supplementation attenuates the fluctuations in the abundances of main phyla in the rumen liquid microbiota (Guo et al., 2024), such effects were not observed in the rumen solids microbiota during SARA challenges. We speculate this could be due to differences in relative abundances of phyla between rumen liquid and solid fractions that made them respond differently to SCFP supplementation (Petri et al., 2013; Brede et al., 2020).

It has been reported that SCFP supplementation increases rumen VFA production, resulting in greater milk yield and feed efficiency in lactating dairy cows (Callaway and Martin, 1997; Hristov et al., 2010; Poppy et al., 2012). Xiao et al. (2016, 2018) found that the SCFP supplementation (XPC) increased ruminal butyrate concentrations and *Butyrivibrio* abundance, a genera within the Lachnospriraceae family. The authors also reported increased richness in rumen liquid microbiota but did not observe any effect on the richness and diversity of rumen solids microbiota in calves. In the current study, we also observed that SCFPa supplementation promoted the growth of *Butyrivibrio* in rumen solids during SARA challenges; however, as reported before, SCFP supplementation did not increase butyrate concentration (Khalouei et al., 2020). Butyrate and propionate provide energy for the rumen epithelial cells; thus, higher concentrations of these VFAs may promote the development of rumen papillae and the absorption area for VFA (Meehan and Beiko, 2014). However, excessive accumulation of these VFA in the rumen will reduce the rumen pH (Gozho et al., 2005). It has been demonstrated that SCFP promotes the length and weight of rumen papillae (Lesmeister et al., 2004; Brewer et al., 2014; Xiao et al., 2016) and stabilizes the rumen pH during high-starch diet feeding or under grain-based SARA conductions (Allen and Ying, 2012; Li et al., 2016; Dias et al., 2018). In agreement with the above, our companion papers (Khalouei et al., 2020; Guo et al., 2022) reported that SCFPb-2X supplementation increased the rumen pH, reduced ruminal propionate and free-LPS concentrations during grain-based SARA challenges, indicating the benefit of SCFP on improving rumen health and reducing the adverse effects of SARA.

Different members of the rumen microbiota utilize substrates different from those in the diet. Thus, maintaining a diverse microbial community during periods of dietary or metabolic stress can improve the efficiency of nutrient utilization (Henderson et al., 2015; Khafipour et al., 2016). Tun et al. (2020) found that SCFP supplementation attenuated the reduction in richness and diversity of the rumen liquid microbiota during the SARA challenge. Similarly, our parallel study showed that the reduction in the evenness of the rumen liquid microbiota following exposure to SARA was attenuated by SCFP supplementation (Guo et al., 2024). However, we did not observe a treatment effect on the alpha diversity of rumen solid microbiota in the current study. We did, however, observe a treatment effect on the beta diversity as determined by PERMANOVA. That being said, the significant treatment effect obtained by PERMDISP analysis, which takes into account dispersion in data, suggests that the observed effect may have been influenced by the differences in the taxonomic composition within each group.

The effect of SCFP supplementation on the functionality of the rumen microbiota was also studied. Nitrogen metabolism in the rumen includes protein degradation that provides amino acids and nitrogen for bacteria and microbial protein synthesis that is the major source of protein for the cow when it reaches the small intestine (Storm and Ørskov, 1983; Clark et al., 1992). Protein degradation in the rumen requires the combination of proteolytic and nonproteolytic enzymes and amino acid uptake by rumen microbes (Bach et al., 2005). Positive effects of amylases on protein degradation have been reported in earlier studies (Assoumani et al., 1992; Tománková and Kopečný, 1995). Bach et al. (2005) reported that the abundance of cellulolytic bacteria decreases while proteolytic bacteria are less affected as rumen pH declines. When fed high-concentrate diets, starch-degrading bacteria predominate in the rumen while the number of cellulolytic bacteria decreases, and as a consequence, fiber and protein degradation reduces (Mould and Ørskov, 1983; Cherdthong et al., 2010). Carbohydrates are the main energy resources for rumen bacteria, and they can also be used as carbon skeletons for microbial protein synthesis. The nitrogen uptake by the ruminal microbes can be improved when the readily fermentable carbohydrates increase (Stern and Hoover, 1979; Casper and Schingoethe, 1989; Cameron et al., 1991). When the rate of carbohydrate fermentation exceeds the protein degradation rate, microbial protein synthesis decreases (Nocek and Russell, 1988). The reduction of ruminal nitrogen metabolism when cows are fed a high-grain diet may be related to the increase in digesta passage rate in the rumen that reduces the proteolytic activity of rumen microbiota (Sullivan and Martin, 1999; Bach et al., 2005).

Several studies have been conducted to determine the effect of SCFP on ruminal nitrogen metabolism. It has been reported that SCFP increases nitrogen utilization by rumen bacteria and reduces ammonia and methane emissions by dairy cows (Hristov et al., 2010). Tun et al. (2020) found that SCFP attenuated the reduction of nitrogen metabolism during the SARA challenge. Our results partly agreed with these previous studies, as we found that SCFP promoted predicted amino acid metabolism and carbohydrate metabolism, including ascorbate and aldarate metabolism, fructose and mannose metabolism, and inositol phosphate metabolism during SARA challenges. Additionally, we also observed that SCFP promoted the colonization of several fibrolytic bacteria, including *Lachnospiraceae UCG-009, Treponema, unclassified Lachnospiraceae*, and *unclassified Ruminococcaceae* during the SARA challenges. As fibrolytic bacteria have a high preference for ammonia (Bryant, 1973), the SCFP supplementation may have increased the nitrogen metabolism in our study.

Microbe-to-microbe association networks offer high-level insights into the changes in the global structure of microbial communities (Peschel et al., 2021). We found that only the SCFPb-2X treatment maintained the characteristics of the network inside the rumen solids microbial community under grain-based SARA challenges, indicating a more resilient and stable microbial community during this treatment. Besides, members from the *Lachnospiraceae NK3A20* group*, Phocaeicola, Anaerotruncus, Anaerofustis, Senegalimassilis*, and *unclassified Lachnospiraceae* played important hub roles in the network derived from the SCFPb-2X treatment during SARA and had strong positive associations with richness and diversity of the microbiota in rumen solids microbiota. It is conceivable that an increased abundance of the above taxa will assist in maintaining the diversity and richness of the microbial community during dietary or metabolic stress periods.

## 5 Conclusions

Repeated grain-based SARA challenges reduced the richness and diversity and changed the β-diversity of the bacterial community in rumen solids. Grain-based SARA challenges also reduced the relative abundance of cellulolytic bacteria, such as members of Fibrobacteres phyla, reduced the interactions among rumen bacteria, and the diversity of hub taxa in solids microbiota. Supplementation with SCFPb increased the positive connectedness among microbial members in the solid fraction and the diversity of hub taxa. In particular, SCFPb-2X maintained the pre-SARA properties of the rumen solids microbial network during the SARA challenges. Grain-based SARA challenges inhibited the growth of several fibrolytic bacteria, while SCFP supplementation also promoted the growth of several fibrolytic taxa during SARA challenges. SARA challenges also reduced the predicted nitrogen metabolism in the microbiota of rumen solids, while SCFP supplementation attenuated this decline, which may prevent the reduction in microbial protein synthesis. Due to the limitations of the 16S rRNA gene sequencing to classify taxa at the species level and limitations of current databases when using CowPi to predict the rumen microbiome functionality, further shotgun metagenomic and metabolomic approaches are needed to better understand the consequences of SARA and benefits of SCFPs on the rumen microbiome.

## Data availability statement

The rumen solids sequencing data were deposited into NCBI Sequence Read Archive (SRA) and are available under the Bioproject ID: PRJNA1139131.

## Ethics statement

All procedures used in this study was approved by the University of Manitoba Animal Care Committee (Protocol # F14-038) and followed the guidelines of the Canadian Council for Animal Care (CCAC, 1993).

## Author contributions

JG: Investigation, Methodology, Formal analysis, Visualization, Writing – original draft. ZZ: Formal analysis, Investigation, Methodology, Writing – review & editing. LG: Formal analysis, Methodology, Writing – review & editing, Resources, Supervision. MZ: Methodology, Writing – review & editing. IY: Writing – review & editing, Conceptualization. EK: Conceptualization, Writing – review & editing, Funding acquisition, Investigation, Methodology, Project administration, Resources, Supervision, Validation. JP: Conceptualization, Funding acquisition, Investigation, Methodology, Project administration, Resources, Supervision, Writing – review & editing, Data curation.
