# Supplementary material for: Postbiotics from Saccharomyces cerevisiae fermentation stabilize rumen solids microbiota and promote microbial network interactions and diversity of hub taxa during grain-based subacute ruminal acidosis (SARA) challenges in lactating dairy cows
Source: Front Microbiol. 2024 Aug 16;15:1409659. doi: 10.3389/fmicb.2024.1409659 (PMC11362103; doi:10.3389/fmicb.2024.1409659)

**Supplementary materials**

**Postbiotics *from Saccharomyces cerevisiae* fermentation stabilize rumen solids microbiota and promote microbial network interactions and diversity of hub taxa during grain-based subacute ruminal acidosis (SARA) challenges in lactating dairy cows**

Junfei Guo, Zhengxiao Zhang, Le Luo Guan, Mi Zhou, Ilkyu Yoon, Ehsan Khafipour, and Jan C. Plaizier

| **Supplementary Table 1**. Effects of treatment (Control, SCFPa, SCFPb-1X, and SCFPb-2X), and stage of SARA on alpha-diversity of rumen solids microbiota | | | | | | | | | | | | | | | | | |
| --- | --- | --- | --- | --- | --- | --- | --- | --- | --- | --- | --- | --- | --- | --- | --- | --- | --- |
| Item | Treatment^1^ | Stage^2^ | | | | | | | | | | | | SEM | *P*-value^3^ | | |
|  |  | wk -4 | wk -1 | wk 1 | wk 3 | wk 4  Pre-SARA1 | wk 5  SARA1/1 | wk 5  SARA1/2 | wk 7  Post-SARA1 | wk 8  SARA2/1 | wk 8  SARA2/2 | wk 10  Post-SARA2 | wk 12  Post-SARA2 |  | Treat | Stage | Treat x Stage |
| Shannon’s diversity | Control | 8.56^a^ | 8.49^a^ | 8.35^acd^ | 8.57^a^ | 8.59^a^ | 7.76^cde^ | 7.63^e^ | 8.46^a^ | 7.71^de^ | 7.22^e^ | 8.61^a^ | 8.44^ac^ | 0.12 | 0.46 | <.0001 | 0.59 |
|  | SCFPa | 8.45^b^ | 8.59^b^ | 8.01^b^ | 8.67^ab^ | 8.78^a^ | 7.21^c^ | 7.34^c^ | 8.65^b^ | 6.84^c^ | 6.98^c^ | 8.53^b^ | 8.39^b^ | 0.12 |  |  |  |
|  | SCFPb-1X | 8.74^a^ | 8.43^ab^ | 8.65^ab^ | 8.29^b^ | 8.41^ab^ | 6.90^c^ | 7.24^c^ | 8.46^ab^ | 7.03^c^ | 7.02^c^ | 8.51^ab^ | 8.34^ab^ | 0.12 |  |  |  |
|  | SCFPb-2X | 8.4339^a^ | 8.52^a^ | 8.63^a^ | 8.36^ab^ | 8.76^a^ | 7.70^bc^ | 7.37^c^ | 8.59^a^ | 7.55^bc^ | 7.42^c^ | 8.56^a^ | 8.69^a^ | 0.13 |  |  |  |
| Pielou’s Evenness | Control | 0.93^a^ | 0.91^a^ | 0.91^a^ | 0.93^a^ | 0.92^a^ | 0.88^b^ | 0.87^b^ | 0.91^a^ | 0.87^b^ | 0.85^b^ | 0.92^a^ | 0.92^a^ | 0.01 | 0.07 | <.0001 | 0.27 |
|  | SCFPa | 0.92^ab^ | 0.93^a^ | 0.87^b^ | 0.93^a^ | 0.93^a^ | 0.82^c^ | 0.85^c^ | 0.93^a^ | 0.81^c^ | 0.83^c^ | 0.91^ab^ | 0.90^ab^ | 0.01 |  |  |  |
|  | SCFPb-1X | 0.93^a^ | 0.92^ab^ | 0.92^ab^ | 0.90^b^ | 0.91^ab^ | 0.81^c^ | 0.85^c^ | 0.91^b^ | 0.82^c^ | 0.85^c^ | 0.91^ab^ | 0.91^ab^ | 0.01 |  |  |  |
|  | SCFPb-2X | 0.92^a^ | 0.93^a^ | 0.93^a^ | 0.94^a^ | 0.92^a^ | 0.88^b^ | 0.86^b^ | 0.93^a^ | 0.86^b^ | 0.84^b^ | 0.93^a^ | 0.93^a^ | 0.01 |  |  |  |
| Faith’s Phylogenetic diversity | Control | 98.38^a^ | 101.96^a^ | 92.88^ab^ | 105.43^a^ | 102.99^a^ | 77.93^bc^ | 71.73^c^ | 97.73^a^ | 77.79^bc^ | 64.65^c^ | 99.68^a^ | 98.50^a^ | 4.09 | 0.92 | <.0001 | 0.50 |
|  | SCFPa | 95.16^a^ | 97.02^a^ | 95.23^a^ | 106.64^a^ | 108.52^a^ | 71.20^b^ | 67.72^b^ | 100.68^a^ | 62.27^b^ | 65.63^b^ | 101.80^a^ | 100.40^a^ | 4.12 |  |  |  |
|  | SCFPb-1X | 108.85^a^ | 100.02^ab^ | 105.91^ab^ | 99.32^ab^ | 99.58^ab^ | 66.64^c^ | 67.00^c^ | 102.86^ab^ | 66.92^c^ | 57.03^c^ | 101.38^ab^ | 91.99^b^ | 4.10 |  |  |  |
|  | SCFPb-2X | 97.77^b^ | 98.93^b^ | 103.13^ab^ | 90.45^bc^ | 115.24^a^ | 76.36^c^ | 69.67^c^ | 98.81^b^ | 77.19^c^ | 77.01^c^ | 99.88^ab^ | 104.46^ab^ | 4.42 |  |  |  |
| Observed  features | Control | 604.25^a^ | 644.09^a^ | 579.75^ab^ | 632.93^a^ | 633.00^a^ | 479.50^bc^ | 439.37^bc^ | 624.25^a^ | 472.37^bc^ | 382.12^c^ | 646.25^a^ | 599.50^a^ | 34.60 | 0.97 | <.0001 | 0.55 |
|  | SCFPa | 590.98^a^ | 623.65^a^ | 576.25^ab^ | 635.93^a^ | 742.62^a^ | 440.88^bc^ | 406.37^c^ | 647.50^a^ | 366.62^c^ | 376.25^c^ | 639.00^a^ | 633.75^a^ | 34.96 |  |  |  |
|  | SCFPb-1X | 694.37^a^ | 598.12^a^ | 687.12^a^ | 616.42^a^ | 594.50^a^ | 381.25^b^ | 396.87^b^ | 644.00^a^ | 386.82^b^ | 329.77^b^ | 666.91^a^ | 562.87^a^ | 34.75 |  |  |  |
|  | SCFPb-2X | 586.86^bc^ | 576.86^bc^ | 627.86^ab^ | 481.13^bcd^ | 751.86^a^ | 453.57^d^ | 407.29^d^ | 619.57^ab^ | 455.86^cd^ | 464.14^cd^ | 600.29^b^ | 660.67^ab^ | 37.45 |  |  |  |
| ^a-e^Means in a row with different superscripts among Stages are different (*p* < 0.05).  ^1^Treatment: Control = 140 g/d ground corn; SCFPa = 14 g/d Diamond V Original XPC mixed with 126g/d ground corn; SCFPb-1X = 19 g/d NutriTek mixed with 121 g/d ground corn; SCFPb-2X = 38 g/d NutriTek mixed with 102 g/d ground corn.  ^2^Stage: SARA was induced during wk 5 (SARA1) and wk 8 (SARA2) after parturition. Rumen samples were taken on d 2 (SARA1/1, SARA1/2) and d 5 (SARA2/1, SARA2/2) during each SARA week. Wk 4 was considered as Pre-SARA1, wk 7 as Post-SARA1, and wk 10 and 12 as Post-SARA2.  ^3^Statistical analyses were conducted on Box-cox-transformed data for Shannon’s diversity, evenness and Observed features, original data for Faith’s Phylogenetic diversity. Presented means are original values prior to transformation. | | | | | | | | | | | | | | | | | |

**Supplementary Figure 1. Longitudinal shifts in Firmicutes and Bacteroidetes proportions in rumen solids microbiota.** Metagenomic Longitudinal Differential Abundance (MetaLonDA) was used to assess the longitudinal changes in rumen solids microbial communities as lactation progressed. The ASV table was normalized using cumulative sum scaling (CSS) transformation. The longitudinal profiles in each group were fitted with a negative binomial smoothing spline. The blue color represents control and red color represents SCFP groups. The significant time intervals were identified when *P* < 0.05 after multiple testing corrections using Benjamini-Hochberg False Discover Rate (FDR) estimation. a, b) Longitudinal shifts in Firmicutes and Bacteroidetes proportions, respectively, in Control vs. SCFPb-2X group. c, d) Longitudinal shifts in Firmicutes and Bacteroidetes proportions, respectively, in Control vs. SCFPb-1X group. e, f) Longitudinal shifts in Firmicutes and Bacteroidetes proportion, respectively, in Control vs. SCFPa group. The study was started from 4 weeks before until 12 weeks after parturition. SARA challenges were conducted on wk 5 and wk 8 and rumen samples were taken weekly but twice during SARA weeks (SARA1/1, SARA1/2, SARA2/1, SARA2/2).


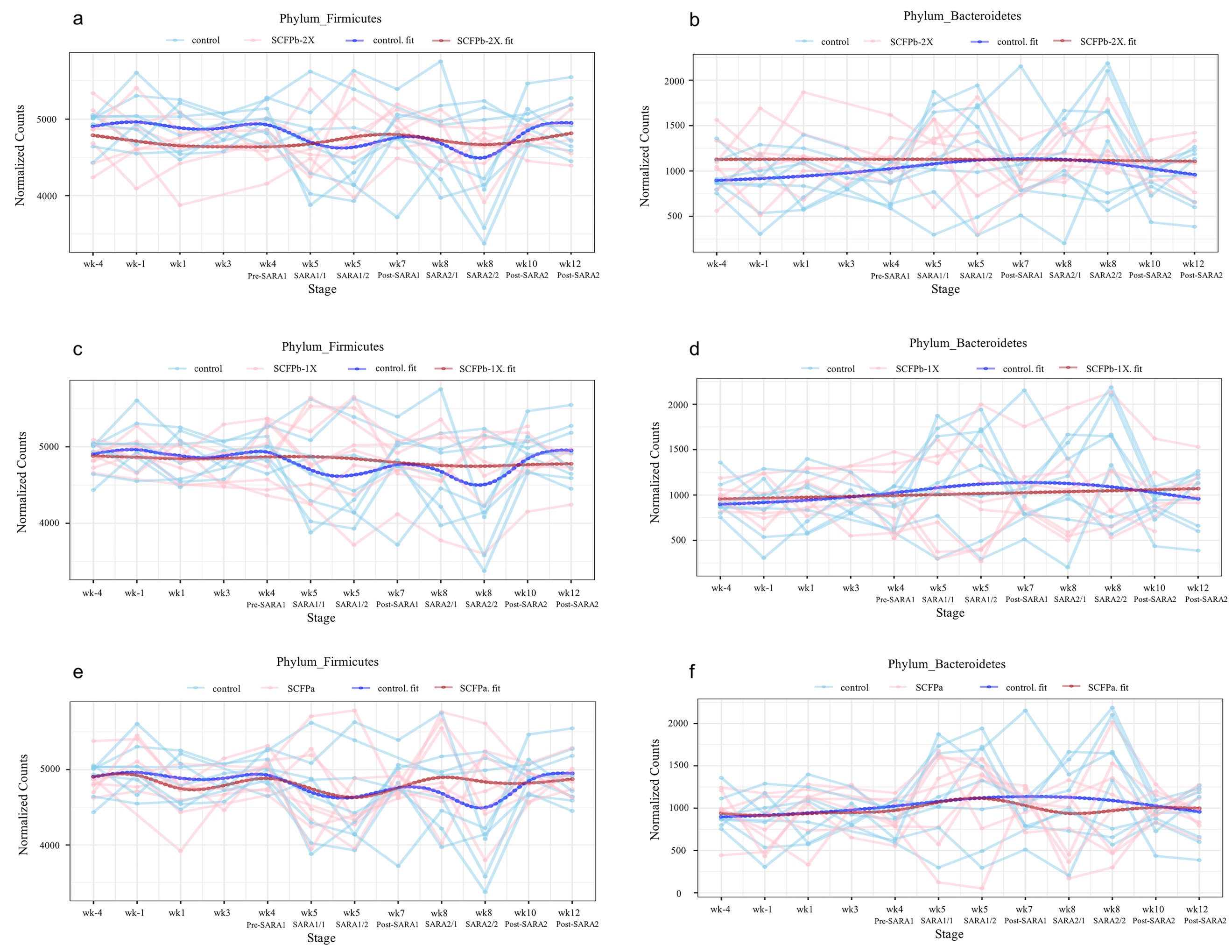


**Supplementary Figure 2.** **Differences in predicted microbial metabolic pathways between non-SARA and SARA stages in control group.** Functionalities of rumen solids microbiota were predicted by CowPi and the results were analyzed by STAMP following log transformation and False Discovery Rate (FDR) correction. Pre-SARA1, Post-SARA1 and Post-SARA2 stages were considered as non-SARA stage, and SARA1/1, SARA1/2, SARA2/1, SARA2/2 stages were considered as SARA stage. Significant differences were considered as *p* < 0.05.


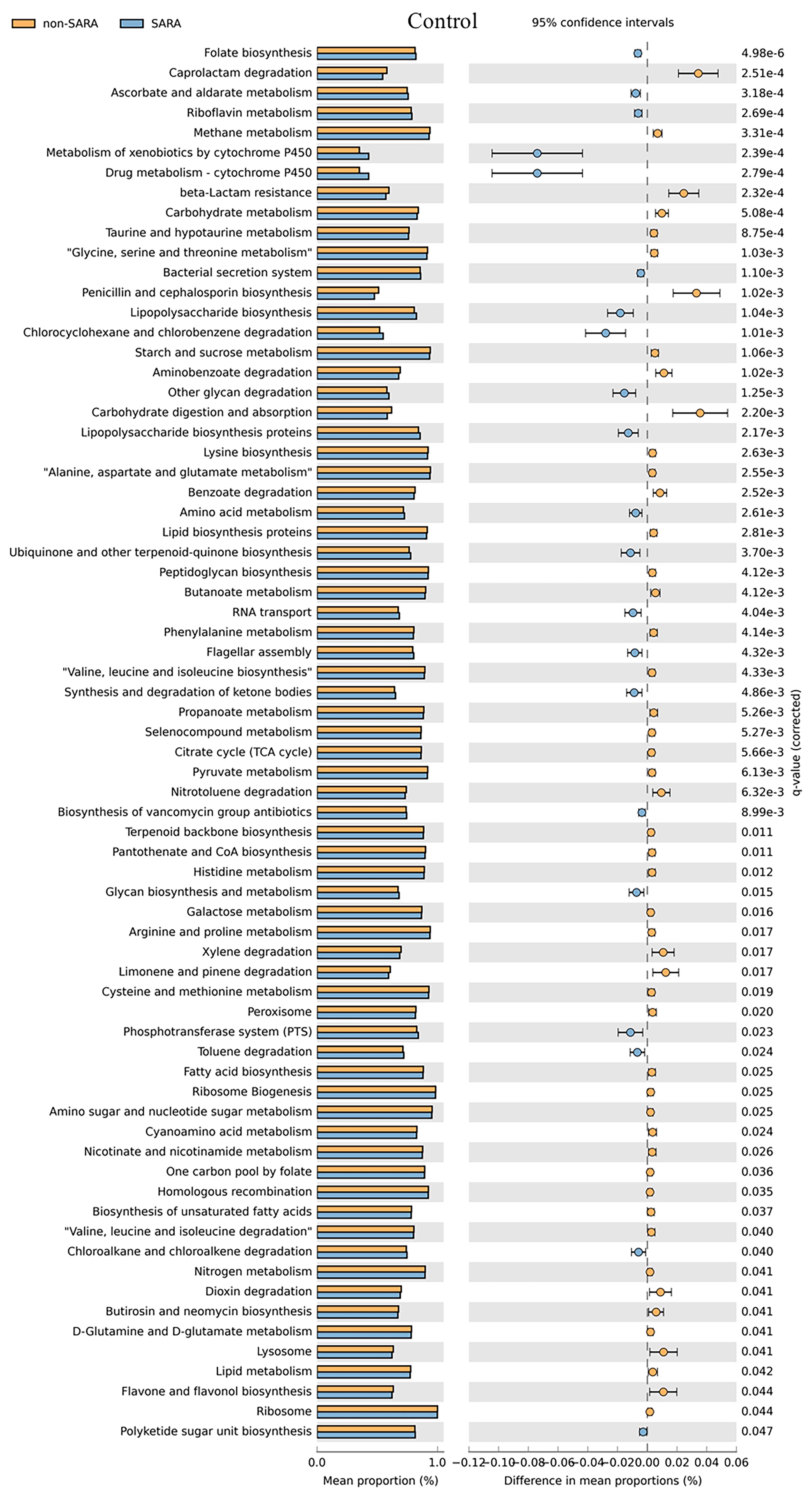


**Supplementary Figure 3.** **Differences in predicted microbial metabolic pathways between non-SARA and SARA stages in SCFPb-2X group**. Functionalities of rumen solids microbiota were predicted by CowPi and the results were analyzed by STAMP following log transformation and False Discovery Rate (FDR) correction. Pre-SARA1, Post-SARA1 and Post-SARA2 stages were considered as non-SARA stage, and SARA1/1, SARA1/2, SARA2/1, SARA2/2 stages were considered as SARA stage. Significant differences were considered as *p* < 0.05.


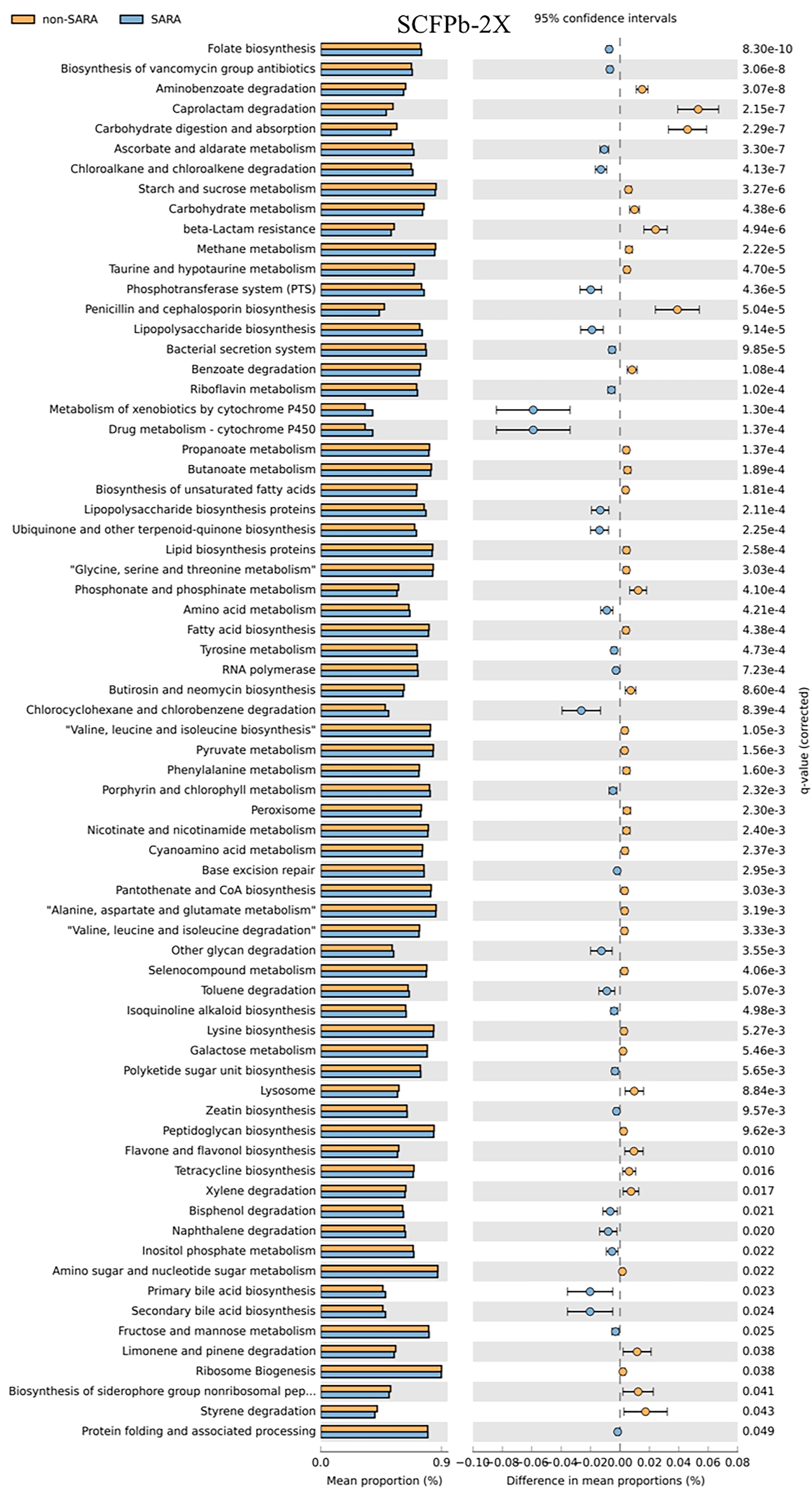


**Supplementary Figure 4.** **Differences in predicted microbial metabolic pathways between non-SARA and SARA stages in SCFPb-1X group**. Functionalities of rumen solids microbiota were predicted by CowPi and the results were analyzed by STAMP following log transformation and False Discovery Rate (FDR) correction. Pre-SARA1, Post-SARA1 and Post-SARA2 stages were considered as non-SARA stage, and SARA1/1, SARA1/2, SARA2/1, SARA2/2 stages were considered as SARA stage. Significant differences were considered as *p* < 0.05.


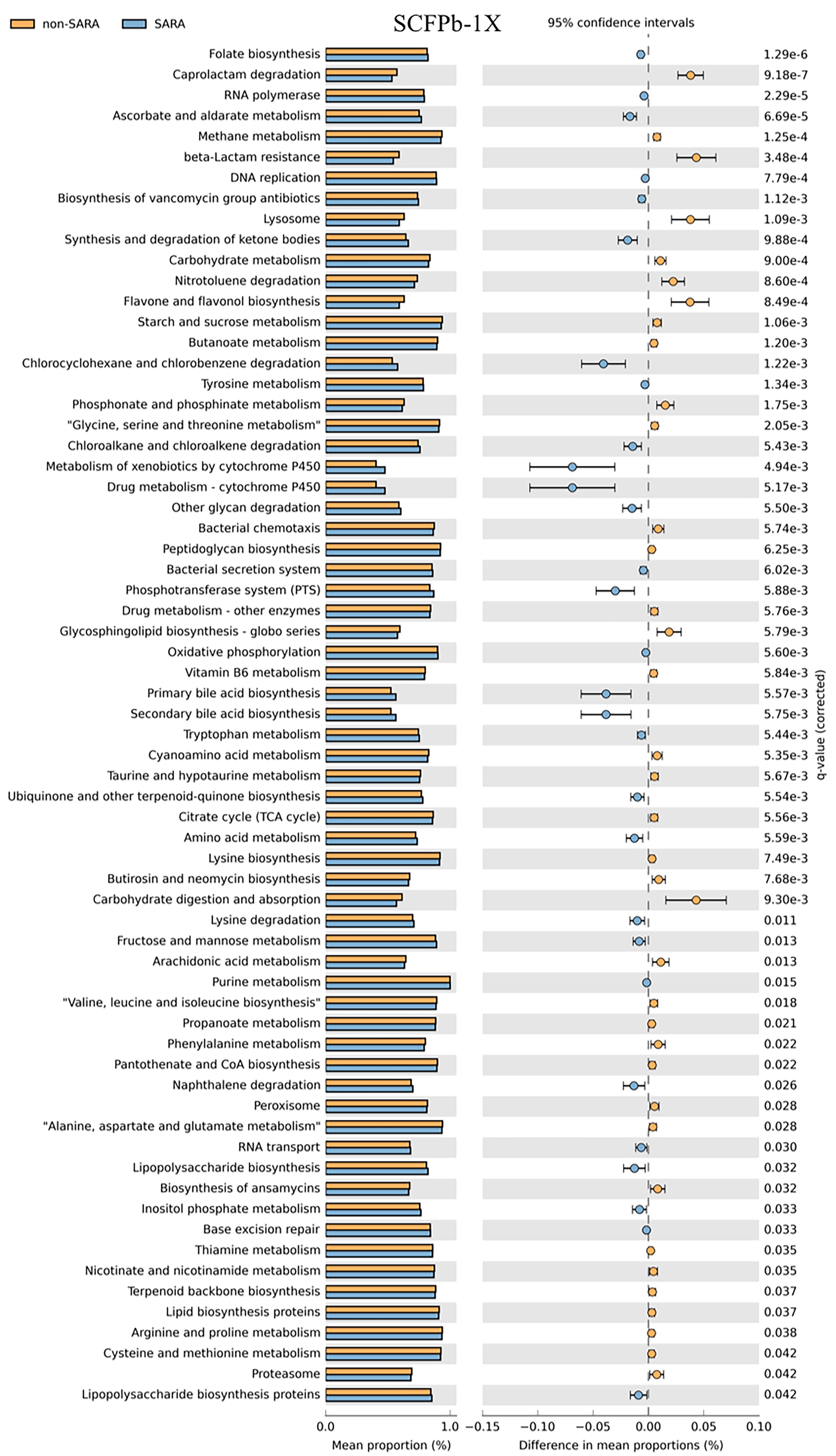


**Supplementary Figure 5. Differences in predicted microbial metabolic pathways between non-SARA and SARA stages in SCFPa group.** Functionalities of rumen solids microbiota were predicted by CowPi and the results were analyzed by STAMP following log transformation and False Discovery Rate (FDR) correction. Pre-SARA1, Post-SARA1 and Post-SARA2 stages were considered as non-SARA stage, and SARA1/1, SARA1/2, SARA2/1, SARA2/2 stages were considered as SARA stage. Significant differences were considered as *p* < 0.05.


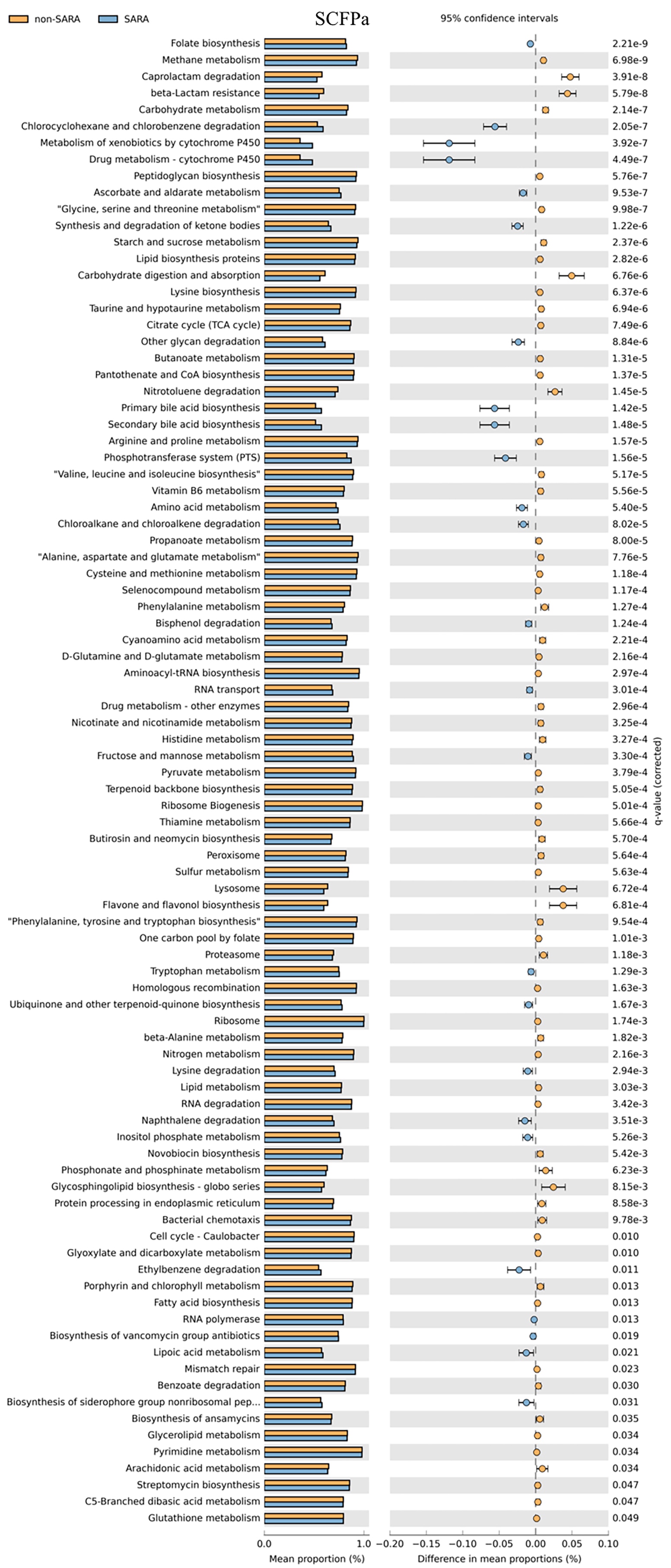


**Supplementary Figure 6.** **Differences in predicted microbial metabolic pathways between Control and SCFPa group during SARA challenges**. Functionalities of rumen solids microbiota were predicted by CowPi and the results were analyzed by STAMP following log transformation and False Discovery Rate (FDR) correction. Significant differences were considered as *p* < 0.05.


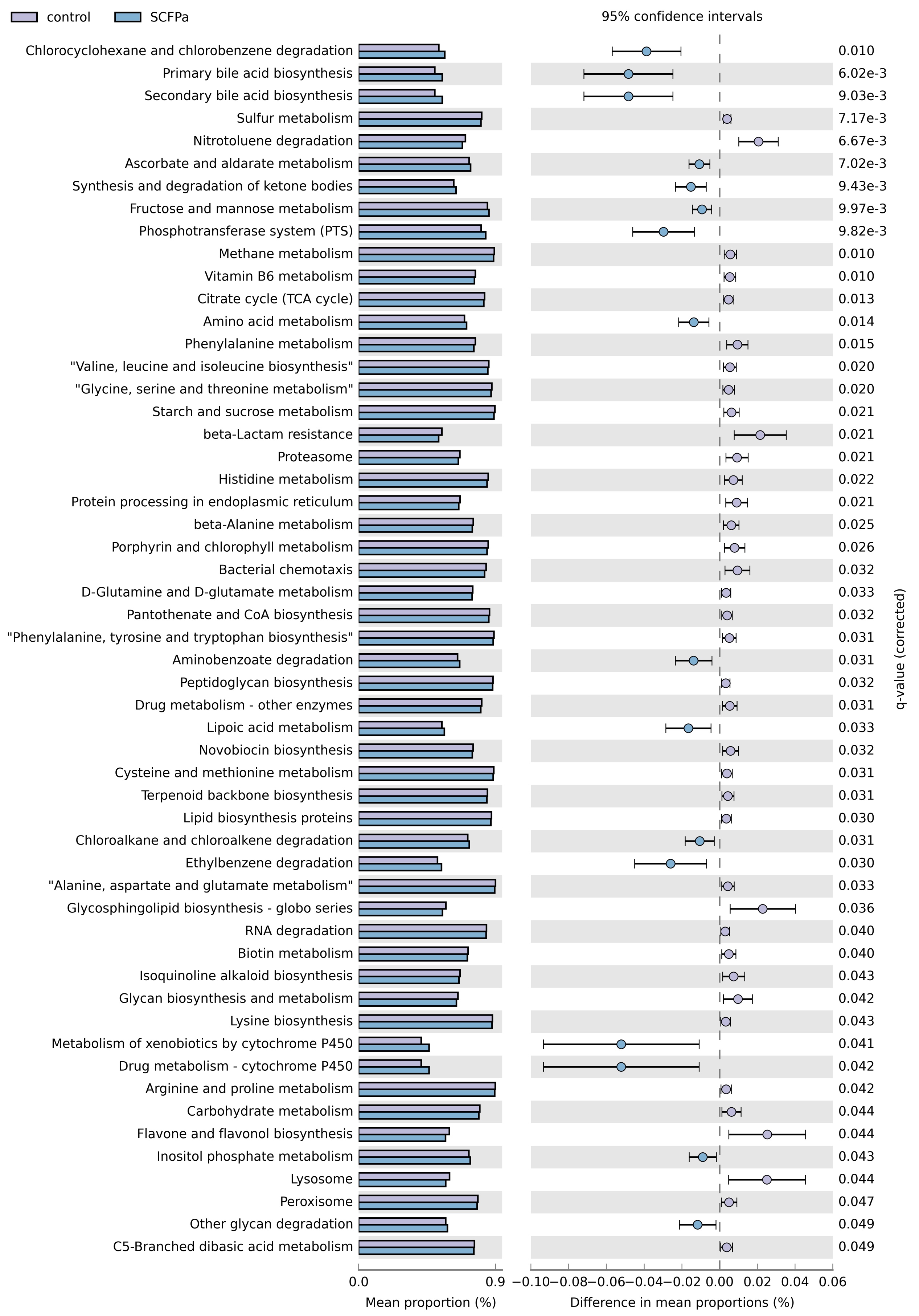

Supplement: Supplementary file 1 [file Data_Sheet_1.docx]
